# Supplementary material for: Reactive aldehyde chemistry explains the missing source of hydroxyl radicals
Source: Nat Commun. 2024 Feb 22;15:1648. doi: 10.1038/s41467-024-45885-w (PMC10883920; doi:10.1038/s41467-024-45885-w)
Supplement: Supplementary file 1 — Supplementary Information [file 41467_2024_45885_MOESM1_ESM.pdf]

Supplementary Information

Reactive Aldehyde Chemistry Explains the Missing Source of Hydroxyl Radicals

Xinping Yang[1, 2, #], Haichao Wang[3, 4, #], Keding Lu[1, *], Xuefei Ma[1], Zhaofeng Tan[1], Bo Long[5], Xiaorui Chen[1], Chunmeng Li[1], Tianyu Zhai[1], Yang Li[1], Kun Qu[1], Yu Xia[5], Yuqiong Zhang[5], Xin Li[1], Shiyi Chen[1], Huabin Dong[1], Limin Zeng[1], Yuanhang Zhang[1, *]

[1] State Key Joint Laboratory of Environmental Simulation and Pollution Control, State Environmental Protection Key Laboratory of Atmospheric Ozone Pollution Control, College of Environmental Sciences and Engineering, Peking University, Beijing, 100871, China.

[2] State Environmental Protection Key Laboratory of Vehicle Emission Control and Simulation, Vehicle Emission Control Center, Chinese Research Academy of Environmental Sciences, Beijing, 100012, China.

[3] School of Atmospheric Sciences, Sun Yat-sen University and Southern Marine Science and Engineering Guangdong Laboratory (Zhuhai), Zhuhai, 519082, China

[4] Guangdong Provincial Observation and Research Station for Climate Environment and Air Quality Change in the Pearl River Estuary, Key Laboratory of Tropical Atmosphere-Ocean System, Ministry of Education, Zhuhai, 519082, China

[5] College of Material Science and Engineering, Guizhou Minzu University, Guizhou, China.

[#] These authors contributed equally to this work.

Correspondence to:
Keding Lu (k.lu@pku.edu.cn), Yuanhang Zhang (yhzhang@pku.edu.cn)

Outline.

Texts

Supplementary Text 1. Detailed information on the warm-season campaigns in China.

Supplementary Text 2. Radical closure experiment.

Supplementary Text 3. Quantum chemical calculations and kinetic methods.

Supplementary Text 4. Sensitivity test for aromatic autoxidation mechanism.

Supplementary Text 5. OH yields from the VOC oxidation through HPC photolysis.

Supplementary Text 6. External model parameters for global campaigns.

Supplementary Text 7. Influences of HAM in ratios of OH_{obs} to OH_{mod} under different NO concentrations in China.

Figures

Supplementary Figure 1. Geographical locations of the seven warm-season campaigns conducted in China.

Supplementary Figure 2. The values and compositions of the modeled k_{OH} in the seven campaigns in China.

Supplementary Figure 3. NO dependence on ratios of observed to modeled OH (OH_{obs}/OH_{mod}) in the seven campaigns in China.

Supplementary Figure 4. The OH production rates from different pathways in two subperiods in the seven campaigns in China.

Supplementary Figure 5. Correlations of missing OH sources with NO and VOCs at low NO conditions in the seven warm-season campaigns in China.

Supplementary Figure 6. The relative enthalpies at 0 K for the H-migration of $R(CO)O_2$ radicals derived from hexanal and the molecules of reactant and transition states for producing conformers.

Supplementary Figure 7. The conceptual scheme of the aromatic autoxidation mechanism.

Supplementary Figure 8. Correlations between the observed and modeled HO_2 concentrations in our seven campaigns.

Supplementary Figure 9. NO dependence on OH_{obs}/OH_{mod} and decline rate of OH_{obs}/OH_{mod} under different sensitivity tests at different NO concentrations in the seven campaigns in China.

Supplementary Figure 10. The relative enthalpies at 0 K for the H-migration of $R(CO)O_2$ radicals derived from acetaldehyde ($CH_3C(O)O_2$).

Supplementary Figure 11. Intrinsic Reaction Coordinate (IRC) for TS1.

Supplementary Figure 12. Intrinsic Reaction Coordinate (IRC) for TS2.

Supplementary Figure 13. Intrinsic Reaction Coordinate (IRC) for TS3.
Supplementary Figure 14. Intrinsic Reaction Coordinate (IRC) for TS4.
Supplementary Figure 15. Intrinsic Reaction Coordinate (IRC) for TS5.
Supplementary Figure 16. The diurnal ambient temperature in the seven warm-season campaigns.
Supplementary Figure 17. The profiles of ALD concentrations and the ratios of propanal to ALD concentrations at the Taizhou, Shenzhen, and Chengdu sites.
Supplementary Figure 18. The profiles of the ratio of butanal versus the sum of butanal and larger R-groups aldehydes at the Taizhou, Shenzhen, and Chengdu sites.

Tables

Supplementary Table 1. Overview of the measurement sites.
Supplementary Table 2. Measured species and information of the instruments in the warm-season campaigns in China.
Supplementary Table 3. Summary of the meteorological parameters and the concentrations of trace gases in the seven warm-season campaigns in China.
Supplementary Table 4. The calculated 1,7 H-migration rate constants of $R(CO)O_2$ radicals derived from hexanal ($CH_3CH_2CH_2CH_2CH_2C(O)O_2$) at different temperatures.
Supplementary Table 5. The reactions and reaction rate constants of the higher aldehyde autoxidation mechanism.
Supplementary Table 6. The φ values achieving perfect agreement between the observed and modeled OH concentrations.
Supplementary Table 7. Values of observed radical concentrations and auxiliary parameters for the noontime during easterly flows during ClearfLo campaign.
Supplementary Table 8. The activation enthalpies for the H-migration of $R(CO)O_2$ radicals derived from acetaldehyde ($CH_3C(O)O_2$) under different methods.
Supplementary Table 9. The relative energies of distinguish conformers for reactant and transition states.
Supplementary Table 10. Vibrational frequency (cm^{-1}) for all structures under different theoretical methods.
Supplementary Table 11. Absolute energies in hartrees.
Supplementary Table 12. Cartesian coordinates (Å) for all reactants and transition states are calculated.

Texts.

Supplementary Text 1: Detailed information on the warm-season campaigns in China

*Location

This study presents seven warm-season (summer and autumn) comprehensive field campaigns, including CareBeijing2006 (Yufa site) and NCP2014 (Wangdu site) campaigns conducted in North China Plain (NCP), PRIDE-PRD2006 (Backgarden site), PRIDE-PRD2014 (Heshan site), and STORM2018 (Shenzhen site) campaigns conducted in Pearl River Delta (PRD), EXPLORE-YRD2018 (Taizhou site) campaign conducted in Yangtze River Delta (YRD), and CHOOSE2019 (Chengdu site) campaign conducted in Szechwan Basin (SCB), as shown in Supplementary Figure 1. Details about the field campaigns can be found in the previous papers [1, 2, 3, 4, 5, 6, 7, 8], and the critical information is summarized in Supplementary Table 1. These sites are located in the four major urban agglomerations in China, all suffering from severe secondary pollution, such as both high ozone (O_3) and particulate matter.

*Instrumentation

As the most critical parameter in the exploration of radical chemistry, concentrations of hydroxyl (OH) and hydroperoxy (HO_2) radicals are measured by the laser-induced fluorescence system (LIF) based on the fluorescence assay by gas expansion technique during all seven campaigns in our study. Besides radical concentrations, a comprehensive set of meteorological and chemical parameters was measured, including the temperature, pressure, relative humidity (RH), photolysis frequency, OH reactivity (k_{OH}), and the concentrations of some essential trace gases (nitric oxide (NO), nitrogen dioxide (NO_2), O_3, volatile organic compounds (VOCs), etc.). Similar instrumentation was deployed at all the sites, and Supplementary Table 2 gives an overview of the instrumentation for these campaigns. Nevertheless, there are several differences among the instrumentations in the seven campaigns. Concentrations of organic peroxy (RO_2) radicals were measured only at the Backgarden, Yufa, Wangdu, and Heshan sites. k_{OH} was measured continuously only at the Backgarden, Yufa, Wangdu, Heshan, Shenzhen, and Chengdu sites. OVOCs were not measured at the Backgarden and Yufa sites. Further instrumental details could be found in previous papers [2, 3, 4, 5, 6, 7, 8, 9].

*Characteristics of air masses

Supplementary Table 3 summarizes the critical meteorological and chemical

parameters during the seven campaigns in China. The ambient temperature in Backgarden was highest, and that in Heshan was lowest, which may be related to the observation seasons. Similarly, the values of $j(O^1D)$ and $j(NO_2)$ measured in Backgarden were the highest among the seven sites. The high temperature in Backgarden corresponded to the high RH, while that in Wangdu corresponded to the low RH. The high temperature and low RH provided good meteorological conditions for the O_3 production in Wangdu. The averaged O_3 concentrations during daytime were the highest in Wangdu (54.0 ppb), followed by Taizhou (46.0 ppb), Yufa (41.4 ppb), Chengdu (40.3 ppb), Backgarden (32.3 ppb), Shenzhen (32.2 ppb), and Heshan (26.5 ppb).

Besides the meteorological conditions, the concentrations of precursors (nitrogen oxide ($NOx = NO + NO_2$) and VOCs) impact O_3 production as well. The alkanes concentrations were highest in Shenzhen, alkenes concentrations were highest in Yufa, and aromatics concentrations were highest in Backgarden. Compared to the VOCs concentrations, reactivity may play a more essential role in O_3 production. Therefore, the combined analysis of VOCs concentration and VOCs reactivity is vital for exploring O_3 prevention. The typical OVOC species, HCHO, is mainly produced as secondary pollutants within the photochemical process. The HCHO concentrations were the highest in Chengdu (7.2 ppb), followed by Wangdu (7.1 ppb), Heshan (5.9 ppb), Taizhou (4.2 ppb), and Shenzhen (3.3 ppb). Further analysis of the meteorological and chemical parameters of the seven campaigns could be found in previous papers [2, 3, 4, 5, 6, 7, 8, 9, 10].

***The total OH reactivity and the speciated analysis**

In the OH experimental budget analysis, the OH destruction rate is calculated as the product of the observed OH concentration and k_{OH}. k_{OH} was observed continuously at the Backgarden, Yufa, Wangdu, Heshan, Shenzhen, and Chengdu sites. Regretfully, k_{OH} was not constantly measured at the Taizhou site. As more and more observed OVOCs are constrained in the model, coupled with the fact that numerous OVOCs have been simulated in the model, the simulated k_{OH} values are getting better and better matched with the measured values [2, 9, 10]. Overall, due to the lack of k_{OH} measurement at the Taizhou site, the impact of missing k_{OH} on missing OH sources is inevitable, while this impact might be relatively small.

Supplementary Figure 2 shows the contributions of carbon monoxide (CO), NOx, primary VOCs (PVOCs), and OVOCs to the modeled k_{OH} in the seven campaigns in China. The inorganic (CO and NOx) reactivity is universally lower than VOCs. The OVOC reactivity accounts for about half of the VOC reactivity,

indicating that OVOCs play a significant role in atmospheric chemistry.

To better explore the effect of chemical species on radical chemistry, we further categorized k_{OH} into k_{NO}, k_{VOCs}, k_{AVOCs}, k_{BVOCs}, and k_{OVOCs}. k_{NO} denotes the NO reactivity, and k_{VOCs} denotes the difference between k_{OH} and inorganic reactivities (CO, NOx, O_3, and SO_2). k_{AVOCs} represent the sum reactivities of alkanes, alkenes, and aromatics. k_{BVOCs} mainly represent the isoprene reactivity. k_{OVOCs} represent the k_{VOCs} minus the sum of k_{AVOCs}, k_{BVOCs}, and formaldehyde (HCHO) reactivity. In this case, k_{OVOCs} denote all known and unknown OVOCs except HCHO. The removal of HCHO from k_{OVOCs} is mainly because the simple structure of HCHO makes the contribution of HCHO to radical chemistry clear.

Supplementary Text 2: Radical closure experiment

The radical closure experiment, which is valuable for testing our current understanding of radical chemistry, is utilized in this study. It is conducted using a zero-dimensional box model with constrained photolysis frequency, meteorological parameters, and critical trace gas concentrations. We considered the Regional Atmospheric Chemistry Mechanism updated version 2 (RACM2) as the base model [5, 11, 12, 13] (using Interactive Data Language version 8.3, IDL 8.3). The uncertainty of the model calculation is a combination of uncertainties in the measurements used as model constraints and reaction rate constants, which is approximately 40% [3, 5].

The schematic diagram of the radical closure experiment was reported by Lu et al. [14]. There are two types of model-observation comparisons: one is the comparison of radical concentrations, and the other is the comparison between radical production and destruction rates [14]. As for the comparison between observed and simulated radical concentrations, a zero-dimensional chemical box model based on RACM2 was utilized in this study. The model was constrained to CO, NO, NO_2, methane (CH_4), O_3, gaseous nitrous acid (HONO), and VOC concentrations and photolysis frequencies, water vapor, ambient temperature, and pressure as well. For RACM2, the measured detailed VOC species were lumped into several categories, which is introduced in previous studies [3, 5]. The hydrogen (H_2) and CH_4 mixing ratio was assumed to be 550 ppb and 1900 ppb, respectively. Other parameters were from the observations with 5-min time resolution, which represents the actual atmospheric environmental conditions every day during campaigns. Given the significance of ambient temperature on the results of quantum chemical calculations, herein, it's worth mentioning that the temperature input in the model was the real ambient temperature observed in campaigns rather than a constant value.

Additionally, a spin-up time of 2 days at the beginning of simulations was used to reach steady-state conditions for long-lived species. A fixed dilution equivalent of 24-h lifetime was added in the model to represent the dry deposition. Based on the above configuration in the model, simulated radical concentrations would be obtained and then they will be compared to the observed concentrations to investigate whether the chemical mechanisms are correct, which is the most direct indication to identify whether the chemical mechanisms in the troposphere are complete or not.

The latter is also called the radical experimental budget, in which all the data must be the observed results, including the radical concentration and OH reactivity. For the seven sites in this study, RO_2 concentrations were not measured at the Backgarden, Yufa, Taizhou and Shenzhen sites, so the HO_2 and RO_2 experimental budget cannot be conducted. As for the OH experimental budget, the production rate is quantified from the primary production rate (HONO photolysis, O_3 photolysis, ozonolysis of alkenes) and secondary production rate (dominated by HO_2 + NO and also a minor part from HO_2 + O_3), as shown in Supplementary Equation (1). The OH destruction rate is directly calculated by the product of the observed OH concentration and the observed k_{OH}, as shown in Supplementary Equation (2) [6, 15]. The OH radical production and destruction rates should be balanced due to their short lifetime, and thus the discrepancy between OH production and destruction rates is interpreted as the missing OH sources.

$$P_{OH} = j_{HONO}[HONO] + \varphi_{OH} j_{O^1D}[O_3] + \sum\{\varphi_{OH}^{i} k_{alkenes+O_3}^{i}[alkenes]_i[O_3]\} + \left(k_{HO_2+NO}[NO] + k_{HO_2+O_3}[O_3]\right)[HO_2]$$

Supplementary Equation (1)

$$D_{OH} = [OH] \times k_{OH}$$

Supplementary Equation (2)

We conducted the OH experimental budget in the seven campaigns comprehensively. For the Taizhou site, we calculated the OH destruction rate by replacing the observed k_{OH} with the modeled k_{OH} because of the lack of continuous measurements of k_{OH}. Thus, the OH destruction rate is the lower limit due to the possible missing k_{OH} at the Taizhou site, and hence, the missing OH sources denoted by the difference between the OH destruction rate and production rate are the lower limit here. Supplementary Figure 4 displays the OH experimental budget at these sites in China, indicating that there are missing OH sources universally at low NO conditions.

Supplementary Text 3: Quantum chemical calculations and kinetic methods

The reactants, transition states, and products were optimized using CCSD(T)-F12a/cc-pVDZ-F12 [16] for the H-migration of $R(CO)O_2$ radicals derived from acetaldehyde ($CH_3C(O)O_2$). Benchmark values are calculated using W3X-L[17]//CCSD(T)-F12a/cc-pVDZ-F12 for $CH_3C(O)O_2$ radicals, as shown in Supplementary Figure 10. We also tested W2X[17]//CCSD(T)-F12a/cc-pVDZ-F12, WMS[18]//M06-2X[19]/MG3S[20], CCSD(T)-F12a/jun-cc-pVTZ//M06-2X/MG3S, CCSD(T)-F12a/cc-pVDZ-F12//M06-2X/MG3S. As a result, WMS//M06-2X/MG3S was in good agreement with our benchmark results, with only a 0.09 kcal/mol difference, as shown in Supplementary Table 8. Therefore, WMS//M06-2X/MG3S method was chosen for investigating the H-migration of $CH_3CH_2CH_2CH_2CH_2C(O)O_2$ radicals. The reaction rate constants were calculated by multi-structural transition state theory [21] with Eckart tunneling [22]. The rate constant is calculated as in Supplementary Equation (3):

$$k_{\mathrm{MS-TST}} = F_{\mathrm{fwd}}^{\mathrm{MS-T}}\, k_{\mathrm{SS-TST}}\, \kappa_{\mathrm{Eckart}} \qquad \text{Supplementary Equation (3)}$$

where $k_{\mathrm{MS-TST}}$ denotes the rate constant for the H-migration of RO_2 radicals, $k_{\mathrm{SS-TST}}$ is the conventional transition state theory rate constant calculated with the WMS//M06-2X/MG3S level, κ_{Eckart} is the tunneling coefficient, and $F_{\mathrm{fwd}}^{\mathrm{MS-T}}$ denotes the torsional anharmonicity factor of the forward reaction [21] which is calculated as in Supplementary Equation (4):

$$F_{\mathrm{fwd}}^{\mathrm{MS-T}} = \frac{F^{\mathrm{MS-T}}(\mathrm{TS})}{F^{\mathrm{MS-T}}(\mathrm{R})} = \frac{Q^{\ddagger-\mathrm{MS-T}}/Q^{\ddagger-\mathrm{SSHO}}}{Q^{\mathrm{R-MS-T}}/Q^{\mathrm{R-SSHO}}} \qquad \text{Supplementary Equation (4)}$$

where $Q^{\ddagger-\mathrm{MS-T}}$ and $Q^{\mathrm{R-MS-T}}$ are the multi-structural partition functions for the saddle points and the reactants, respectively. $Q^{\ddagger-\mathrm{SSHO}}$ and $Q^{\mathrm{R-SSHO}}$ are the single-structural harmonic-oscillator rovibrational partition functions for the lowest-energy saddle point and the reactant, respectively. $k_{\mathrm{SS-TST}}$ and κ_{Eckart} were calculated by the conventional transition state theory [23] with Eckart tunneling [22] based on WMS//M06-2X/MG3S level. The conformational structures of reactants and transition states for $F_{\mathrm{fwd}}^{\mathrm{MS-T}}$ were calculated by a multi-structural method with coupled torsional-potential anharmonicity (MS-T) [24]. TS1, TS2, TS3, TS4, and TS5 correspond to 1, 7 H-migration, 1,6 H-migration, 1,8 H-migration, 1,5 H-migration, and 1,4 H-migration processes as described in Supplementary Figure 6a, respectively. We calculated the relative enthalpies at 0 K for the different H-migration pathways of $CH_3CH_2CH_2CH_2CH_2C(O)O_2$ radicals, in which the 1,7 H-migration was the most favorable pathway in all possible H-shift reactions, as shown in Supplementary Figure 6a. Additionally, we calculated the unimolecular rate constant of 1,7 H-

migration at different temperatures and it is 0.321 s^{-1} at 298 K, as shown in Supplementary Table 4. Here, we used Eckart for tunneling [22], which is often overestimated at low temperatures [25]; this may lead to the values (Supplementary Table 4) overestimated at low temperatures. However, field measurements were done at room temperature or higher. In this study, our campaigns were conducted in summer or autumn without low temperatures, especially the effect of RO_2 autoxidation on radicals focused on noontime and afternoon. To determine whether the temperature was so low that it would overestimate tunneling probability, herein, we present the diurnal ambient temperature in the seven campaigns in Supplementary Figure 16. The daytime temperature varied from 295 K to 307 K, and the variations of temperature during 10:00-16:00 local time were 303-306 K, 299-303 K, 300-304 K, 298-300 K, 298-301 K, 299-301 K, and 300-306 K at the Backgarden, Yufa, Wangdu, Heshan, Taizhou, Shenzhen, and Chengdu sites, respectively. Therefore, the present results are still reliable.

For the reactant $CH_3CH_2CH_2CH_2CH_2C(O)O_2$, we considered five rotatable bonds that are C1-O6, C1-C3, C3-C8, C8-C11, and C11-C14, which generate 237 initial guess conformers by using MSTor 2017 program [26, 27]. These initial conformers were firstly optimized by B3LYP[28]/6-31G(d,p) method. As a result, there are only 70 distinguishable conformers from B3LYP/6-31G(d,p) calculated results. Furthermore, the 70 distinguishable conformers were reoptimized by using M06-2X/MG3S method to lead to the formation of 63 distinguishable conformers. However, for the transition state TS2, there is only one rotatable bond C11-C14, and 3 initial guess conformers were generated and optimized by using B3LYP/6-31G(d,p) method to obtain 3 distinguishable conformers. For the transition state TS4, there are two rotatable bonds C8-C11 and C11-C14, and 9 initial guess conformers were generated and optimized by using B3LYP/6-31G(d,p) method to obtain 8 distinguishable conformers. For the transition state TS5, there are three rotatable bonds C3-C8, C8-C11, and C11-C14, and 26 initial guess conformers were generated and optimized by using B3LYP/6-31G(d,p) method to obtain 21 distinguishable conformers. There are no rotatable bonds for TS1 and TS3. The molecules of reactant and transition states for generating conformers are plotted in Supplementary Figure 6b. The relative energies of the distinguishable conformer of the reactants and transition states are listed in Supplementary Table 9. The conformers shown in Supplementary Figure 6a are the lowest energy structures for both the reactant and transition states. Extra information on vibrational frequency, absolute energies, and cartesian coordinates are shown in Supplementary Tables 10-12.

We also did intrinsic reaction coordinate (IRC) [29, 30, 31] calculations to show

that the transition state connects with the corresponding reactant and intermediate product as listed in Supplementary Figures 11-15. However, in Supplementary Figure 6a, the reactant and intermediate product are both the lowest conformers of the reactants and intermediates. The lowest conformers can facilely convert to the specific conformer along internal rotation, which is connected with the corresponding transition state.

Supplementary Text 4: Sensitivity test for aromatic autoxidation mechanism

Previous studies have reported that OH regeneration is possible by the autoxidation mechanism of aromatics, including toluene, o-xylene, m-xylene, and p-xylene [32, 33, 34, 35]. To comprehensively evaluate the radical regeneration pathways, we conducted the sensitivity test for aromatic autoxidation mechanism mainly based on the reactions proposed by Wang et al. by theoretical and experimental investigations [34]. The bicyclic peroxy radicals (BPRs), the important intermediates of the OH-initiated oxidation of aromatics, could undergo autoxidation and subsequently produce radicals. The aromatic autoxidation process can be fast enough to compete with the RO_2 bimolecular reactions with HO_2 or NO under atmospheric conditions. This mechanism consists of three H-migration processes. BPRs could undergo the first H-migration and produce another set of peroxy radicals. Next, the ortho- and para-products could undergo another H-migration. Thirdly, the para-products could undergo the third H-migration. Radicals are produced during the H-migration process and the possibly photolysis of hydroxy-hydroperoxides.

The conceptual scheme is displayed in Supplementary Figure 7. Our results demonstrated that the impact of the aromatics autoxidation mechanism on radical chemistry is negligible.

Supplementary Text 5: OH yields from the VOC oxidation through HPC photolysis

To further explore the effects of generalized RO_2 autoxidation on radical chemistry, we made a rule of thumb assumption named Reactive Aldehyde Mechanism (RAM) scheme, in which RO_2 from some certain VOCs (alkanes, alkenes, aromatics, etc.) could undergo autoxidation process similar to that of RO_2 from aldehydes in Higher Aldehyde Mechanism (HAM) scheme. In the RAM scheme, specific VOC classes were set to generate HPC according to Table 1 of Bianchi et al. [36]. Here, the specific VOCs include HC5 (alkanes,

esters, and alkynes with OH rate constant (298 K, 1 atm) between 3.4×10^{-12} and 6.8×10^{-12} cm^3 s^{-1}), HC8 (alkanes, esters, and alkynes with OH rate constant (298 K, 1 atm) greater 6.8×10^{-12} cm^3 s^{-1}), OLT (terminal alkenes), OLI (internal alkenes), DIEN (butadiene and other anthropogenic dienes), BEN (benzene), TOL (toluene), XYM (m-xylene), XYP (p-xylene), XYO (o-xylene), ISO (isoprene) in RACM2.

In this scheme, we defined a simple index, named as φ, to characterize the generalized yield of OH regenerated by the oxidation of VOC through the HPC chemistry, which is derived from an inversed modeling method, to achieve a good agreement between the modeled and observed OH radicals. The definition of φ is shown as follows (Supplementary Equation (5)).

$$\varphi = \Sigma_{j=1}^{n}(\Sigma_{i=1}^{n}A_{ij}\times HPC_j)\times B_j)\times R_j \qquad \text{Supplementary Equation (5)}$$

A_{ij} denotes the yields of different HPC_j species from the oxidation of different VOC_i species.

B_j denotes the yields of OH radicals in photolysis of different HPC_j species.

R_j denotes the ratio of the HPC_j photolysis frequency to ten times MACR photolysis frequency. Ten times MACR photolysis frequency order of about 10^{-4} s^{-1}, which is the base case setup of HPC photolysis rate used in the HAM.

Where φ depends on the values of A_{ij}, B_j and R_j. The HPC species and the HPC yield are heavily dependent on VOC species (A_{ij}, referred to Table 1 of Bianchi et al. [36]). Subsequently, in the HPC photolysis reaction, different HPC species would have different OH yields (B_j) as well as the photolysis frequencies (R_j). For example, the photolysis frequencies may differ by more than a factor of 4 between different HPC species (referred to Table 2 of Liu et al. [37]). The three coefficients would be highly varied in different campaigns, which would probably further amplify the differences in the derived φ in different campaigns.

Herein, based on the reversed model, we calculated the φ value, which is the equivalent mean value for each campaign, in our fourteen campaigns in Supplementary Table 6.

Supplementary Text 6: External model parameters for global campaigns

Besides our seven warm-season campaigns in China, we also explored the influence of HAM mechanism in radical chemistry in some external field campaigns, including PROPHET, GABRIEL, OP3-I, PMTACS-NY2001, IMPACT-L, BEARPEX09, and ClearfLo campaigns. The model parameters of our own seven campaigns are from the observations in the field campaigns.

For the PROPHET, GABRIEL, OP3-I, PMTACS-NY2001, IMPACT-L, and BEARPEX09 campaigns, we refer to the external model parameters in the Supplementary Information in Rohrer et al. [38]. For the ClearfLo campaign, we relied on the data within the original publications, as shown in Supplementary Table 7.

Supplementary Text 7: Influences of HAM in ratios of OH_{obs} to OH_{mod} under different NO concentrations in China

To further explore the influence of HAM in ratios of the observed to modeled OH concentrations (OH_{obs}/OH_{mod}) under different NO concentrations, we present the NO dependence of OH_{obs}/OH_{mod} at two sensitivity tests involving without and with HAM in the model, as shown in Supplementary Figure 9a. The incorporation of HAM into the model caused the decrease of OH_{obs}/OH_{mod}, indicating that a better agreement between the observed and modeled OH concentrations would be achieved when HAM was considered in the model. Additionally, the decline rates of OH_{obs}/OH_{mod} with HAM compared to OH_{obs}/OH_{mod} without HAM at different NO bins are presented in Supplementary Figure 9b. The decline rates for our seven warm-season campaigns, varying from 0-20% at different NO bins, show an increasing trend with the decrease in NO concentrations.

Figures.

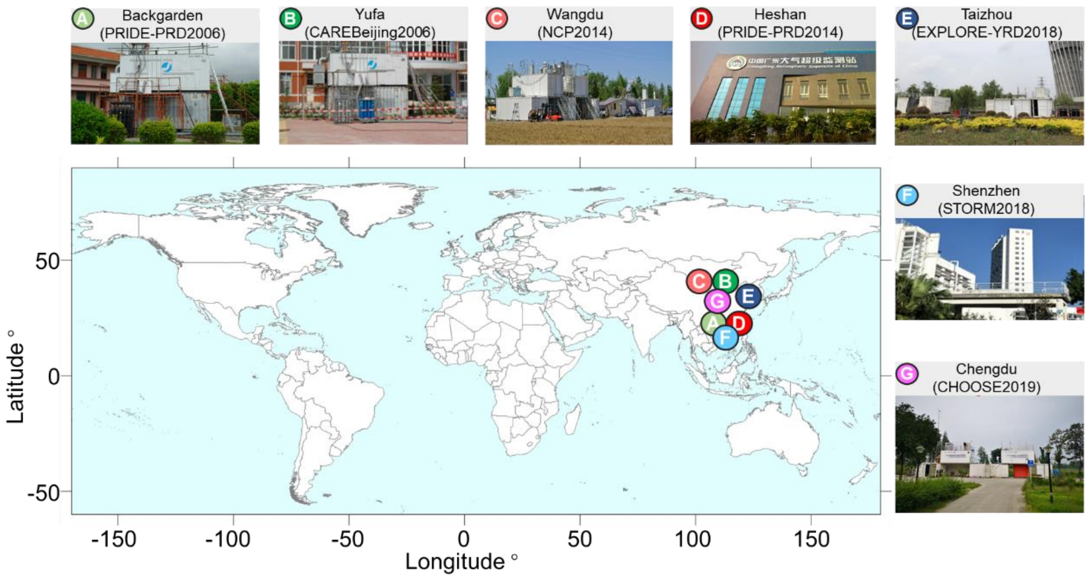

Supplementary Figure 1. Geographical locations of the seven warm-season campaigns conducted in China. Surrounding environmental conditions are shown around the map. The basemap is from the Resource and Environment Science and Data Center, Chinese Academy of Sciences (https://www.resdc.cn/data.aspx?DATAID=205).

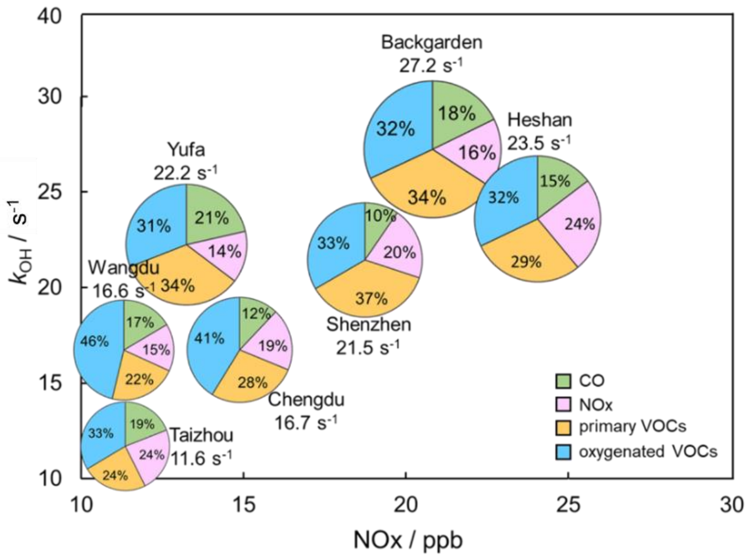

Supplementary Figure 2. The values and compositions of the modeled k_{OH} according to the NOx concentration and k_{OH} in the seven campaigns in China.

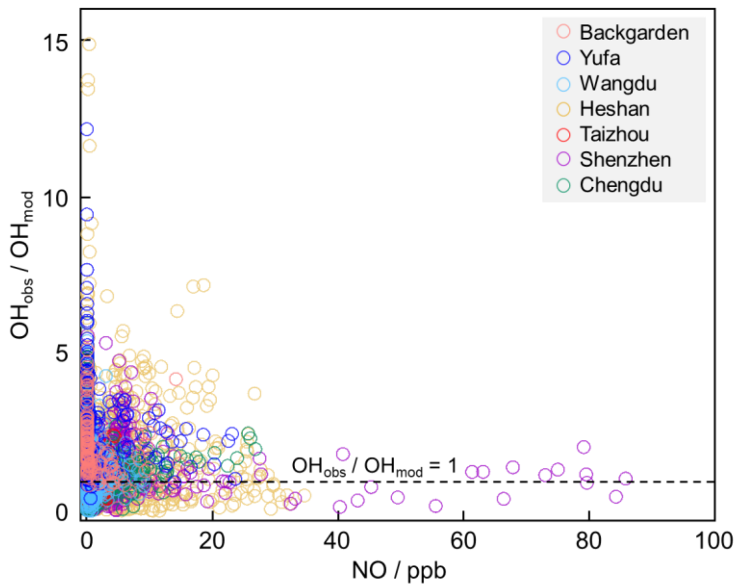

Supplementary Figure 3. NO dependence on ratios of observed to modeled OH (OH_{obs}/OH_{mod}) in the seven campaigns in China.

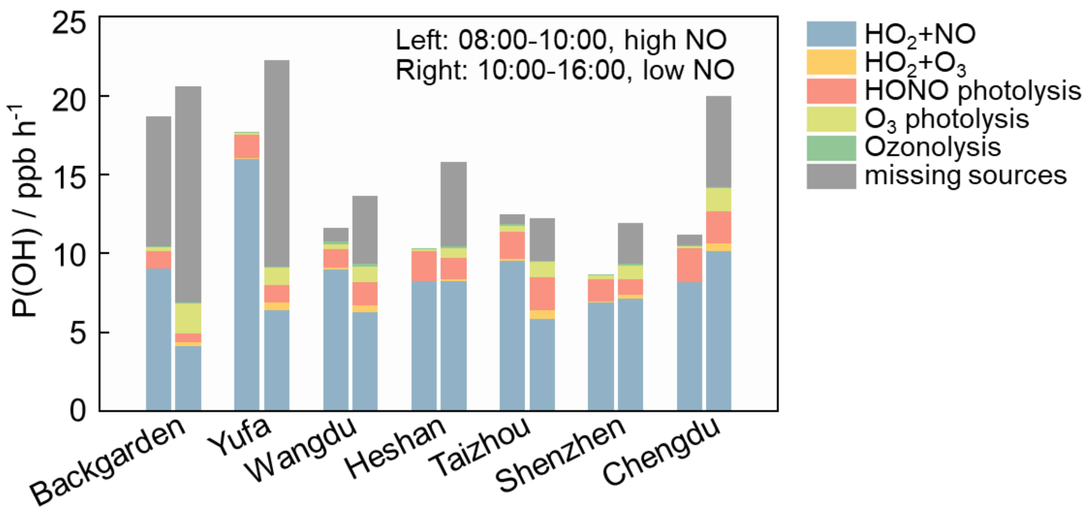

Supplementary Figure 4. The OH production rates (P(OH)) from different pathways in two subperiods (08:00-10:00 local time with high-NO levels and 10:00-16:00 local time with low-NO levels) in the seven campaigns in China.

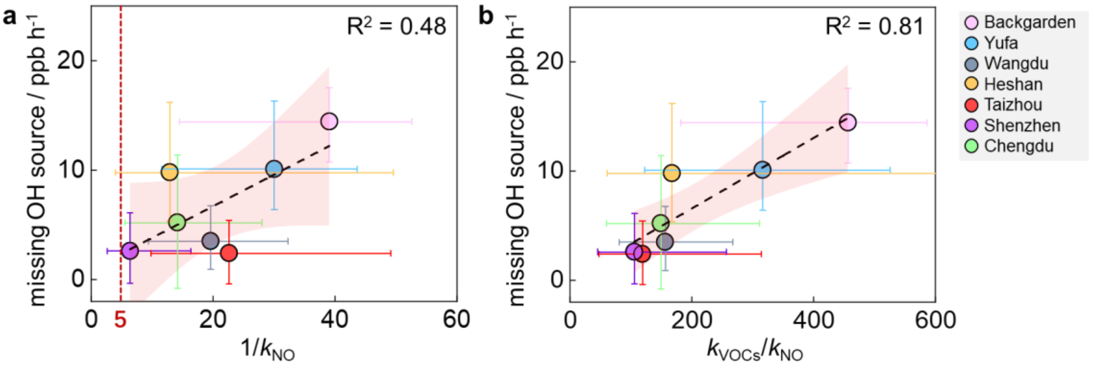

Supplementary Figure 5. Correlations of missing OH sources with the **(a)** inverse of NO reactivity ($1/k_{NO}$), and **(b)** ratios of VOC reactivity versus NO reactivity (k_{VOCs}/k_{NO}) at low NO conditions (10:00-15:00 local time) in the seven warm-season campaigns in China. The circles denote the median values, and the error bars denote the 25th to 75th percentiles. The dotted line indicates the linear fitting curve. The shading shows the 95% confidence intervals.

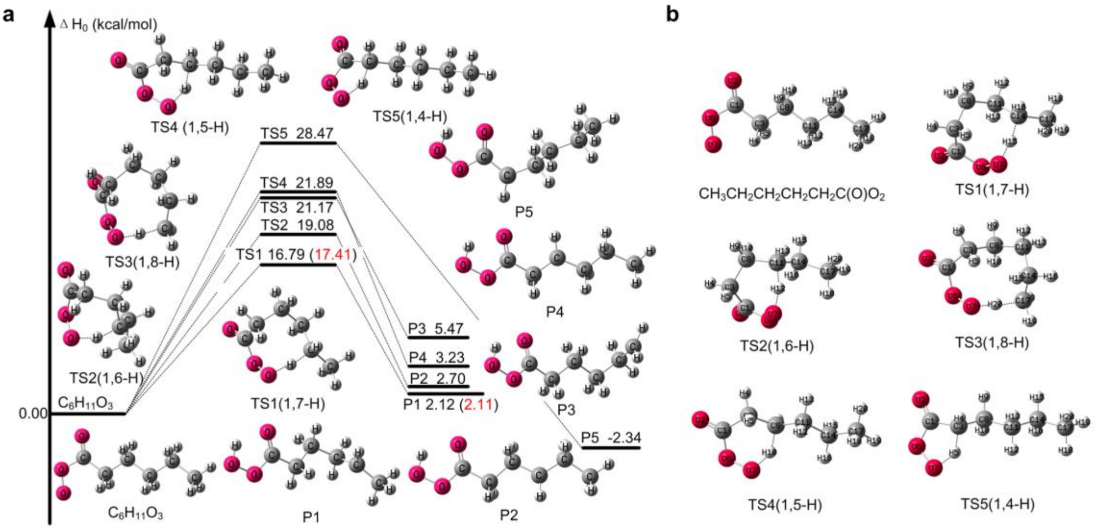

Supplementary Figure 6. (a) The relative enthalpies at 0 K for the H-migration of $R(CO)O_2$ radicals derived from hexanals ($CH_3CH_2CH_2CH_2CH_2C(O)O_2$). Values (black) are given for all species as calculated by M06-2X/MG3S, and values in parentheses (red) are calculated by WMS//M06-2X/MG3S. It is noted that the conformers for reactants, transition states, and intermediate products are the lowest conformer, respectively. **(b)** The molecules of reactant and transition states for producing conformers.

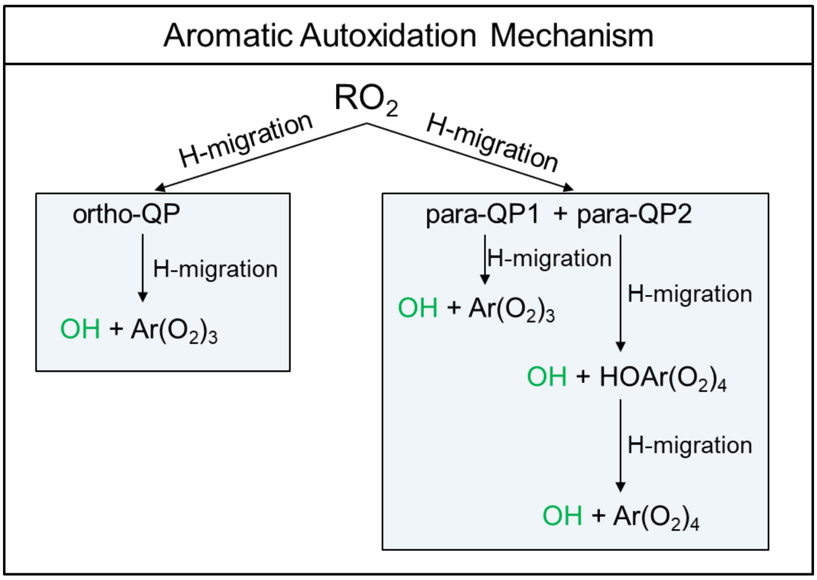

Supplementary Figure 7. The conceptual scheme of the aromatic autoxidation mechanism [34].

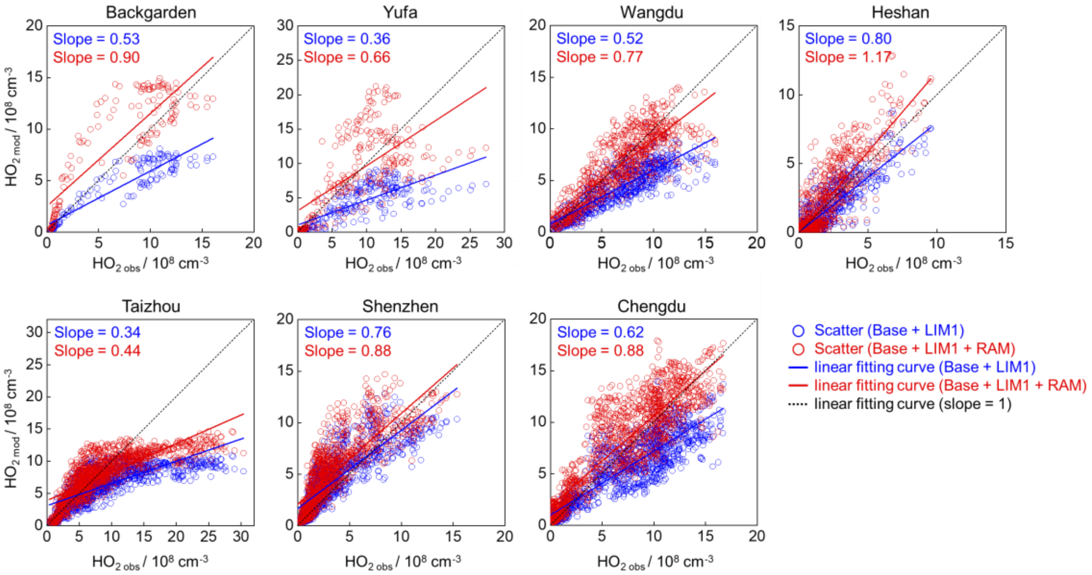

Supplementary Figure 8. Correlations between the observed and modeled HO_2 concentrations in our seven warm-season campaigns. The modeling results include two scenarios, one is the model incorporated with Leuven Isoprene Mechanism updated version 1 (LIM1) (blue color) and the other is the model incorporated with LIM1 and Reactive Aldehyde Mechanism (RAM) together (red color). Only daytime values and NO concentration above the detection limit of the instrument were chosen.

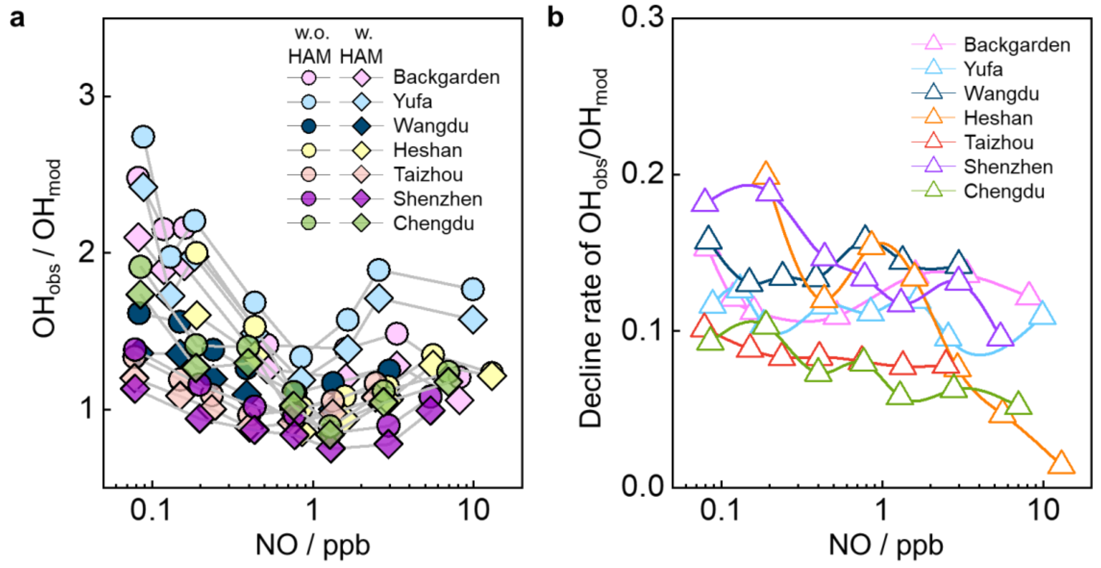

Supplementary Figure 9. (a) NO dependence on ratios of OH_{obs}/OH_{mod} under different sensitivity tests at different NO concentrations in the warm-season campaigns in China during daytime (around 08:00-15:00 local time). HAM denote the Higher Aldehyde Mechanism. Sensitivity test 1: 'w.o.HAM' denotes that modeled OH concentrations are from the model without HAM. Sensitivity test 2: 'w.HAM' denotes that modeled OH concentrations are from the model with HAM. **(b)** Decline rates of OH_{obs}/OH_{mod} with HAM compared to OH_{obs}/OH_{mod} without HAM at different NO concentrations in the warm-season campaigns in China during daytime (around 08:00-15:00 local time).

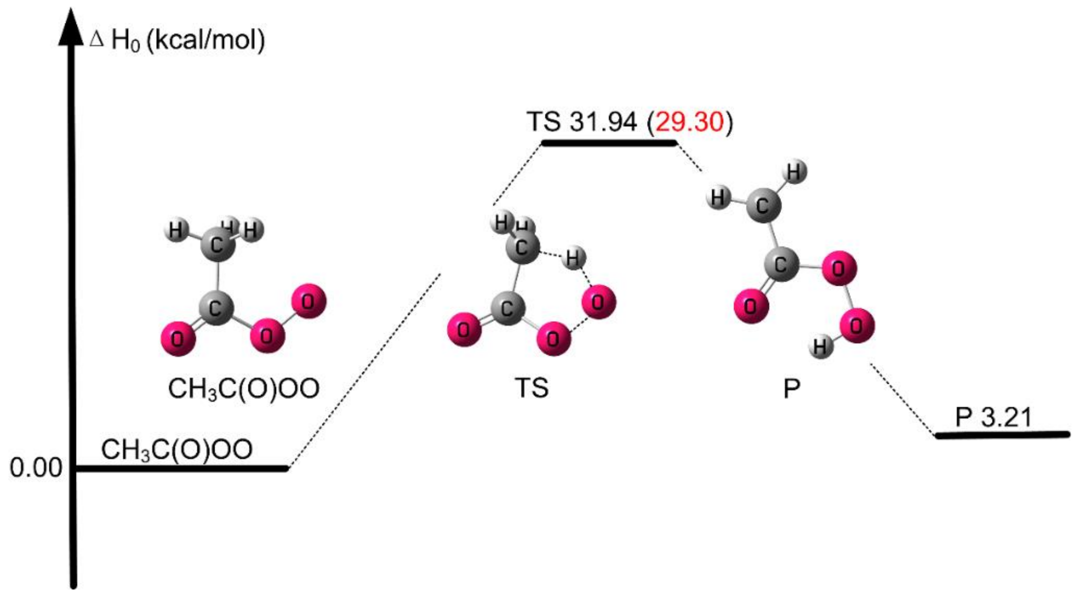

Supplementary Figure 10. The relative enthalpies at 0 K for the H-migration of $R(CO)O_2$ radicals derived from acetaldehyde ($CH_3C(O)O_2$). Values (black) are given for all species as calculated by M06-2X/MG3S, and values in

parentheses (red) are given for transition state (TS) as calculated by W3X-L//CCSD(T)-F12a/cc-pVDZ-F12.

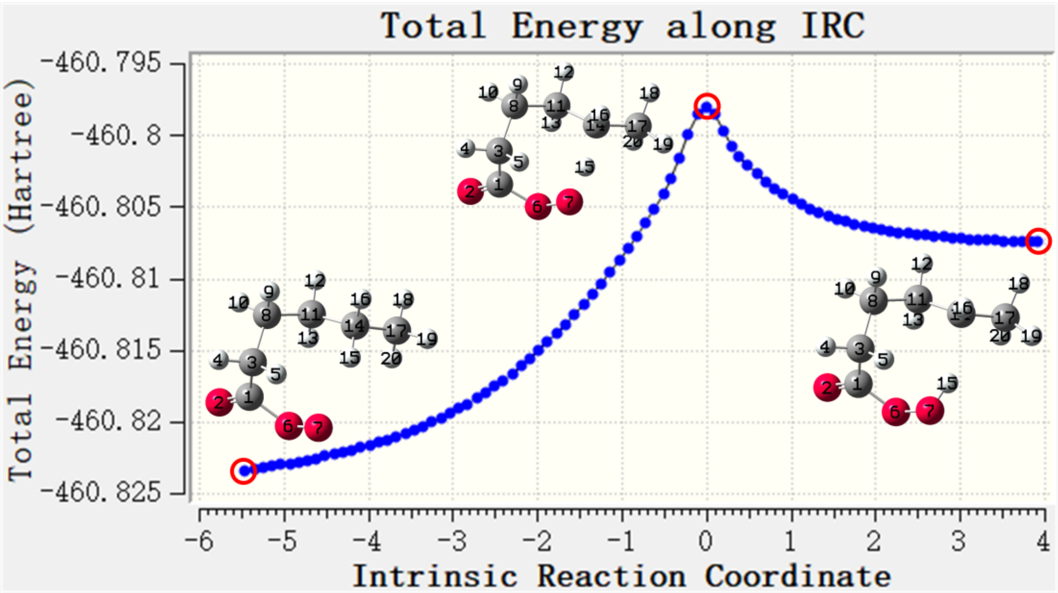

Supplementary Figure 11. Intrinsic Reaction Coordinate (IRC) for TS1 (1,7-H migration).

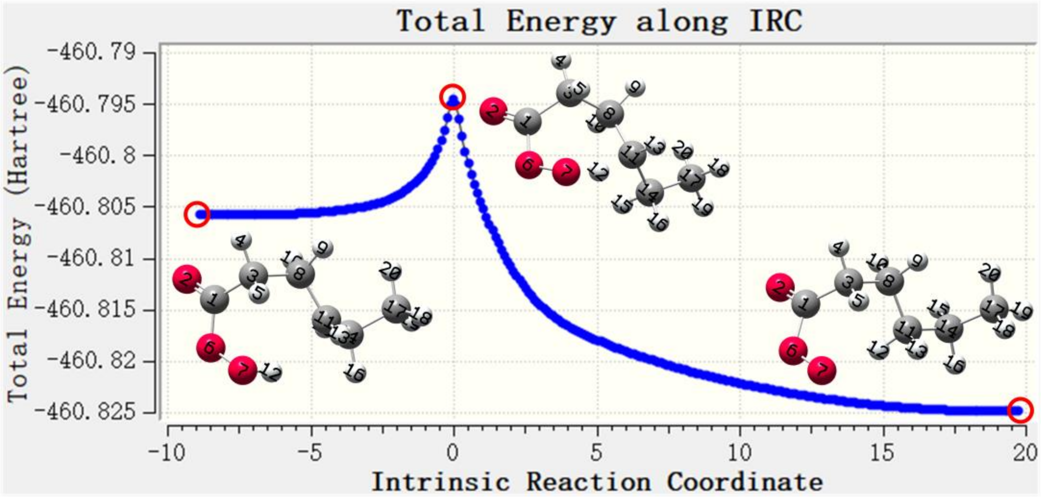

Supplementary Figure 12. Intrinsic Reaction Coordinate (IRC) for TS2 (1,6-H migration).

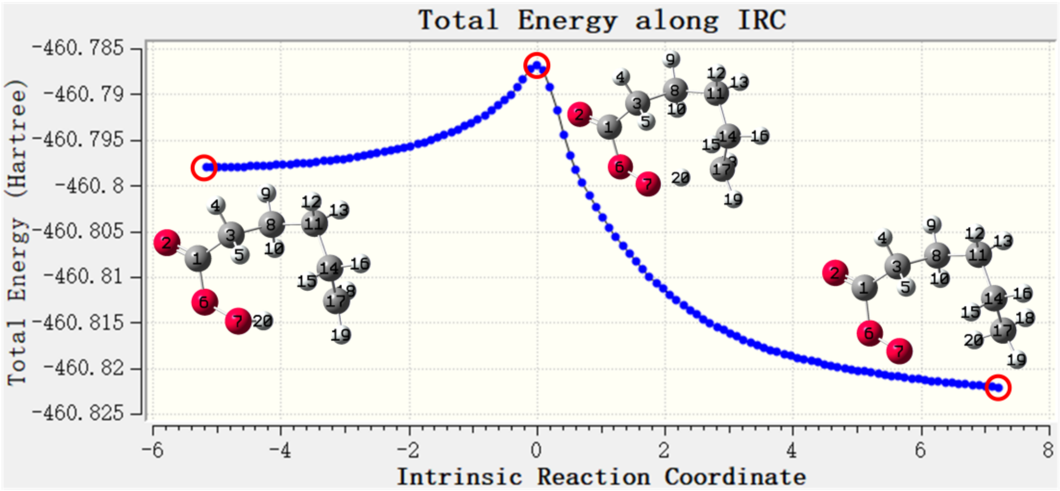

Supplementary Figure 13. Intrinsic Reaction Coordinate (IRC) for TS3 (1,8-H migration).

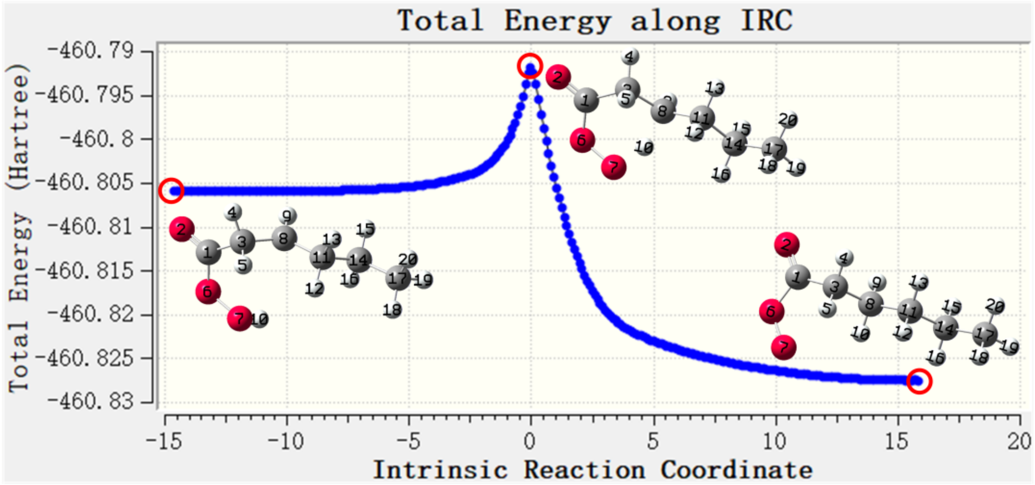

Supplementary Figure 14. Intrinsic Reaction Coordinate (IRC) for TS4 (1,5-H migration).

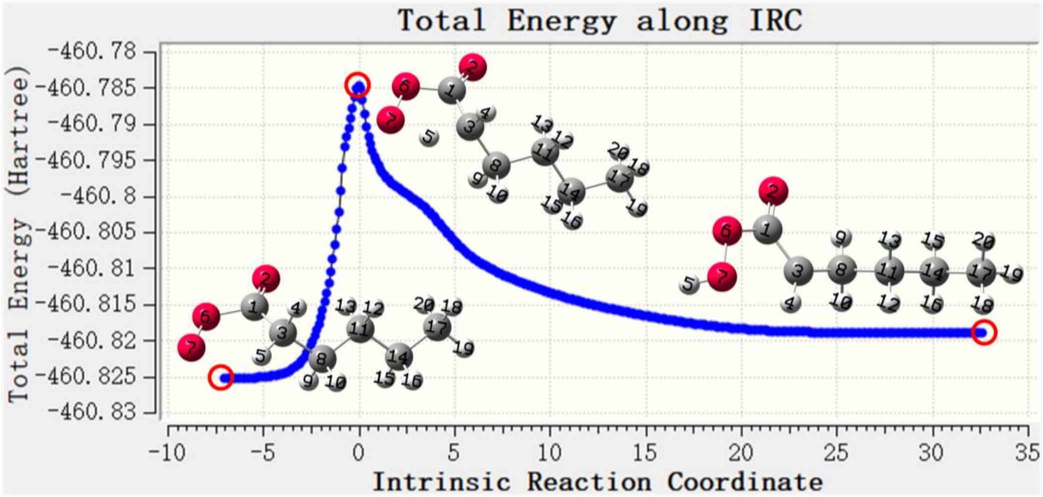

Supplementary Figure 15. Intrinsic Reaction Coordinate (IRC) for TS5 (1,4-H migration).

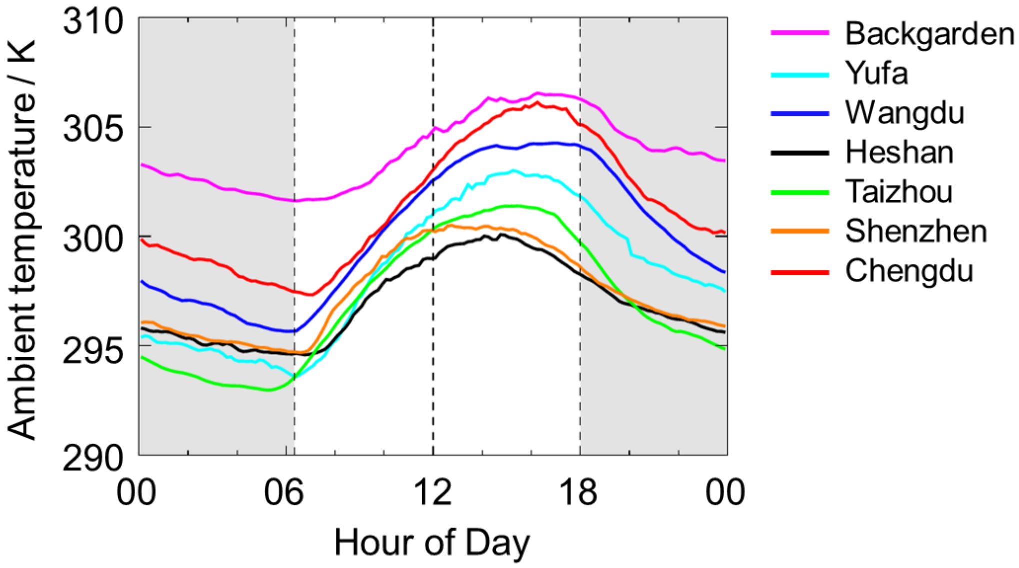

Supplementary Figure 16. The diurnal ambient temperature in the seven warm-season campaigns.

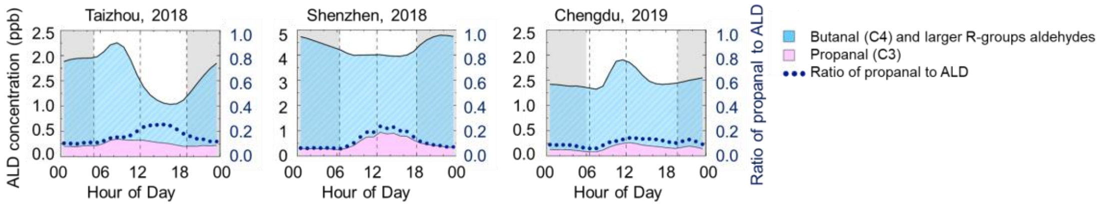

Supplementary Figure 17. The profiles of ALD (propanal, butanal, and larger R-groups aldehydes) concentrations and the ratios of propanal to ALD concentrations at the Taizhou, Shenzhen, and Chengdu sites. The concentrations of propanal are the observed values, and the ALD concentrations are the modeled values because only propanal, butanal, pentanal, and hexanal were measured at these sites. The propanal, butanal, pentanal, and hexanal measurements were complete at the Taizhou, Shenzhen, and Chengdu sites, so we take only these three campaigns as examples here.

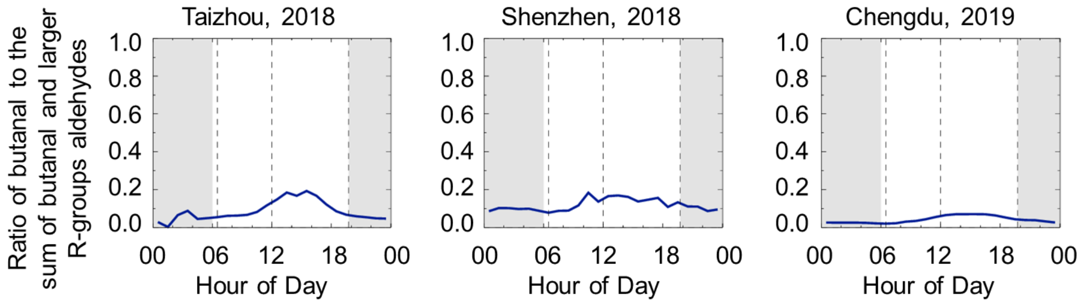

Supplementary Figure 18. The profiles of the ratio of butanal versus the sum of butanal and larger R-groups aldehydes at the Taizhou, Shenzhen, and Chengdu sites. The concentrations of butanal were the observed values, and the concentration of the larger R-groups aldehydes were the modeled values because only propanal, butanal, pentanal and hexanal were measured at these sites. The propanal, butanal, pentanal, and hexanal measurements were complete at the Taizhou, Shenzhen, and Chengdu sites, so we take only these three campaigns as examples here.

Tables.

Supplementary Table 1. Overview of the measurement sites.

Site	Location	Year, season	Period
Backgarden	23.5°N, 113.0°E, PRD	2006, summer	05 Jul.-27 Jul. 2006[*]
Yufa	39.5°N, 116.3°E, NCP	2006, summer	18 Aug.-07 Sep. 2006[#]
Wangdu	38.7°N, 115.2°E, NCP	2014, summer	08 Jun.-08 Jul. 2014
Heshan	22.7°N, 112.9°E, PRD	2014, autumn	19 Oct.-22 Nov. 2014
Taizhou	32.3°N, 119.9°E, YRD	2018, summer	23 May-17 Jun. 2018
Shenzhen	22.6°N, 113.9°E, PRD	2018, autumn	05 Oct.-28 Oct. 2018
Chengdu	30.4°N, 103.8°E, SCB	2019, summer	10 Aug.-25 Aug. 2019

Note that:

[*] Given the continuity of radical measurements, only the period from 20 Jul. to 21 Jul. was be selected to explore the radical chemistry in this study.

[#] The days with southerly (19, 23,27, and 31 Aug. 2006) were analyzed in this study to be consistent with Lu et al. (2013)[2].

Supplementary Table 2. Measured species and information of the instruments in the warm-season campaigns in China.

Parameters	Measurement technique	Time resolution	Detection limit [a]	Accuracy
OH	LIF [b]	30 s	5.0×10^{5} cm^{-3}	±11%
HO_2	LIF [b,c]	30 s	1.0×10^{7} cm^{-3}	±15%
RO_2	PERCA, LIF [d]	5 min	2.0×10^{7} cm^{-3}	±20%
k_{OH}	LP-LIF [e]	5 min	(1.5-2.0) s^{-1}	±10%
Photolysis frequencies	Spectroradiometer	20 s	[f]	±10%
O_3	UV photometry	60 s	500 pptv	±5%
NO	Chemiluminescence	60 s	60 pptv	±20%
NO_2	Chemiluminescence [g]	60 s	300 pptv	±20%
HONO	LOPAP [k]	300 s	10 pptv	±20%
CO	Non-dispersive infrared photometry	60 s	1 ppbv	±1 ppbv
SO_2	Pulsed UV fluorescence	60 s	100 pptv	±10%
HCHO	MaxDOAS, Hantzsch fluorimetry [p]	60 s	25 pptv	±5%
VOCs [m]	GC-MS/FID [n]	1 h	(20-300) pptv	±(15-20)%

Note that:

[a] Signal-to-noise ratio = 1. [b] Laser-Induced Florescence. [c] Chemical conversion via NO reaction before detection. [d] RO_2 concentrations were measured by LIF instrument for Wangdu, Heshan, and Chengdu sites, the other sites missed the measurement of RO_2. [e] Laser Photolysis-Laser-Induced Florescence. [f] Process-specific, 5 orders of magnitude lower than maximum at noon. [g] Photolytic conversion to NO before detection. [k] Long-path absorption photometry. [p] The HCHO measurement was made by a MaxDOAS instrument for Backgarden and Yufa sites while by Hantzsch technique for all the other sites. [m] C_2-C_{12} VOCs. [n] Gas Chromatography with Mass Spectrometry/with Flame Ionization Detection.

Supplementary Table 3. Summary of the meteorological parameters and the concentrations of trace gases (08:00-17:00 averaged) in the seven warm-season campaigns in China. The $j(O^1D)$ and $j(NO_2)$ are averaged values for noontime (11:00-13:00).

Parameters	Backgarden	Yufa	Wangdu	Heshan	Taizhou	Shenzhen	Chengdu
temperature (°C)	30.8	25.2	27.0	21.9	23.8	24.3	29.1
pressure (hPa)	1002.9	1003.9	999.5	1010.1	1007.8	1009.7	940.6
RH (%)	72.3	72.0	58.0	66.0	70.7	75.4	64.0
$j(O^1D)$ (10^{-5} s^{-1})	3.6	2.0	1.7	1.3	1.9	1.8	2.0
$j(NO_2)$ (10^{-3} s^{-1})	7.6	6.4	5.7	4.1	7.3	5.7	6.6
CO (ppb)	948.6	913.6	577.6	642.7	416.1	386.6	425.9
NO (ppb)	5.7	2.1	1.0	3.6	1.1	2.6	3.7
NO_2 (ppb)	14.3	10.4	9.8	18.7	9.5	14.9	11.4
O_3 (ppb)	32.3	41.4	54.0	26.5	46.0	32.2	40.3
HONO (ppb)	1.0	0.6	0.9	1.4	0.6	0.5	1.1
alkanes (ppb)	13.9	13.9	8.3	16.7	10.1	20.2	10.1
alkenes (ppb)	2.1	7.3	3.2	6.0	3.0	2.8	2.7
aromatics (ppb)	11.2	7.4	2.6	8.6	2.6	8.2	3.9
HCHO (ppb)	--	--	7.1	5.9	4.2	3.3	7.2

Supplementary Table 4. The calculated 1,7 H-migration rate constants of $R(CO)O_2$ radicals derived from hexanal ($CH_3CH_2CH_2CH_2CH_2C(O)O_2$) at different temperatures.

Temperature (K)	k_{SS-TST} (s^{-1})	κ_{Eckart}	F_{fwd}^{MS-T}	k_{MS-TST} (s^{-1})
200	7.28×10^{-8}	1.83×10^{5}	0.09	1.20×10^{-3}
220	3.75×10^{-6}	1.43×10^{4}	0.08	4.05×10^{-3}
240	9.99×10^{-5}	2.03×10^{3}	0.06	1.30×10^{-2}
260	1.61×10^{-3}	4.57×10^{2}	0.06	4.06×10^{-2}
280	1.74×10^{-2}	1.47×10^{2}	0.05	1.23×10^{-1}
298	1.13×10^{-1}	6.64×10^{1}	0.04	3.21×10^{-1}
320	8.32×10^{-1}	3.16×10^{1}	0.04	9.95×10^{-1}
340	4.10×10^{-1}	1.90×10^{1}	0.03	2.64×10^{-0}

Note that:

k_{SS-TST} is the conventional transition state theory rate constant calculated with the WMS//M06-2X/MG3S level. κ_{Eckart} is the tunneling coefficient. F_{fwd}^{MS-T} denotes the torsional anharmonicity factor of the forward reaction. k_{MS-TST} is the rate constant for the 1,7 H-migration of RO_2 radicals.

Supplementary Table 5. The reactions and reaction rate constants of the higher aldehyde autoxidation mechanism.

No.	Reaction	Reaction rate constant
No. 1	$R(CO)O_2 \rightarrow \cdot OOR(CO)OOH$	0.321 s^{-1}
No. 2	$\cdot OOR(CO)OOH + NO \rightarrow \cdot OR(CO)OOH + NO_2$	8.7×10^{-12} cm^3 s^{-1}
No. 3	$\cdot OR(CO)OOH \rightarrow HOR(CO)OO\cdot$	1.15×10^{6} s^{-1}
No. 4	$HOR(CO)OO\cdot \rightarrow HOR(CO)OOH$	0.9 s^{-1}
No. 5	$HOR(CO)OOH \rightarrow HO_2 + HPC$	10^4 s^{-1}
No. 6	HPC + *hv* → OH + ACO_3 + HKET	10×*j*MARC

Note that:

- ACO_3 and HKET denote acetyl peroxy radicals and hydroxy ketone, respectively. *j*MACR represents the photolysis rate constant of methacrolein (MACR).
- The rate constant of No. 1 is from our quantum chemical calculations.
- The rate constant of No. 2 is from Table 1 of Tan et al.[6].
- The reactions of Nos. 3-5 are from Wang et al.[39] and the rate constants of both No. 3 and No. 4 are from Wang et al.[39].
- As for No. 5, Wang et al.[39] did not give the rate constant directly. However, as mentioned by Wang et al.[39], the RO radicals could react with O_2 at effective rates of (0.5~5) $\times10^4$ s^{-1} with rate coefficients of 10^{-15}~10^{-14} cm^3 molecules^{-1} s^{-1} according to the studies from Atkinson[40]. The radical in No. 5 is α-hydroxy radicals (R_1R_2COH), and Atkinson[41] once reported the reactions of O_2 and the simplest α-

hydroxy radicals (CH_2OH) with the $k(CH_2OH+O_2)$ is 9.0×10^{-12} cm^3 $molecules^{-1}$ s^{-1} at 298 K, which is larger than the rate constant of $RO+O_2$. The rate coefficients of CH_2OH+O_2 reactions reported in previous studies were summarized on the IUPAC website (https://iupac-aeris.ipsl.fr/show_datasheets.php?category=Gas-phase+organics%3A+R_oxygen) and were all higher those that of $RO+O_2$ reactions. To obtain a conservative result, we chose the rate constant in No. 5 as 10^4 s^{-1}.

- The photolysis rate of No. 6 is a conservative result from Liu et al.[37].

Supplementary Table 6. The φ values achieving optimized agreement between the observed and modeled OH concentrations.

Sites (Campaigns)	φ	Ambient temperature (K)
Backgarden (PRIDE-PRD2006)	2.2	306.5[a]
Yufa (CAREBeijing2006)	2.2	303.0[a]
Wangdu (NCP2014)	1.5	304.2[a]
Heshan (PRIDE-PRD2014)	1.1	300.1[a]
Taizhou (EXPLORE-YRD2018)	1.4	301.4[a]
Shenzhen (STORM2018)	0.4	300.5[a]
Chengdu (CHOOSE2019)	1.6	306.0[a]
Michigan (PROPHET)	2.6	298.1[b]
Suriname (GABRIEL_daytime)	2.93	296.1[c]
Suriname (GABRIEL_afternoon)	2.6	296.1[d]
Borneo rainforest (OP3-I)	1.75	300.1[e]
New York City (PMTACS-NY2001)	0.68	301.7[f]
Tokyo (IMPACT-L)	0.6	304.3[f]
California (BEARPEX09)	0.2	299.1[g]
London (ClearfLo)	0.45	300.6[h]

Note that:

[a] Max diurnal temperature for noontime

[b] Mean temperature for the time window 10:00-11:00 am

[c] Mean temperature for daytime (08:00-17:00)

[d] Mean temperature for afternoon (14:00-17:00)

[e] Mean temperature for noon time (11:00-12:00)

[f] Mean temperature for noon time (11:00-13:00)

[g] Mean temperature for daytime (09:00-15:00)

[h] Max noontime temperature during easterly flow

Supplementary Table 7. Values of observed radical concentrations and auxiliary parameters for the noontime during easterly flows during ClearfLo campaign.

Parameters	Mean Values	Remark
O_3 (ppb)	87.8	Max noontime concentration during easterly flow in Table 3 of Whalley et al. (2018)[42]
NO (ppb)	11.9	Max noontime concentration during easterly flow in Table 3

		of Whalley et al. (2018)[42]
NO_2 (ppb)	39.3	Max noontime concentration during easterly flow in Table 3 of Whalley et al. (2018)[42]
HONO (ppb)	0.89	Max noontime concentration during easterly flow in Table 3 of Whalley et al. (2018)[42]
CO (ppb)	311	Max noontime concentration during easterly flow in Table 3 of Whalley et al. (2018)[42]
CH_4 (ppb)	1971.5	Max noontime concentration during easterly flow in Table 3 of Whalley et al. (2018)[42]
ETH (ppb)	6	Max noontime concentration during easterly flow in Table 3 of Whalley et al. (2018)[42]
HC3 (ppb)	9.7	Max noontime concentration during easterly flow in Table 3 of Whalley et al. (2018)[42]
HC5 (ppb)	4.2	Max noontime concentration during easterly flow in Table 3 of Whalley et al. (2018)[42]
HC8 (ppb)	5.1	Max noontime concentration during easterly flow in Table 3 of Whalley et al. (2018)[42]
CLX (ppb)	0.12	Max noontime concentration during easterly flow in Table 3 of Whalley et al. (2018)[42]
ETE (ppb)	1.7	Max noontime concentration during easterly flow in Table 3 of Whalley et al. (2018)[42]
DIEN (ppb)	0.02	Max noontime concentration during easterly flow in Table 3 of Whalley et al. (2018)[42]
LIM (ppb)	0.23	Max noontime concentration during easterly flow in Table 3 of Whalley et al. (2018)[42]
OLI (ppb)	0.14	Max noontime concentration during easterly flow in Table 3 of Whalley et al. (2018)[42]
OLT (ppb)	0.57	Max noontime concentration during easterly flow in Table 3 of Whalley et al. (2018)[42]
BEN (ppb)	0.3	Max noontime concentration during easterly flow in Table 3 of Whalley et al. (2018)[42]
TOL (ppb)	1.44	Max noontime concentration during easterly flow in Table 3 of Whalley et al. (2018)[42]
XYM (ppb)	0.72	Max noontime concentration during easterly flow in Table 3 of Whalley et al. (2018)[42]
XYO (ppb)	0.03	Max noontime concentration during easterly flow in Table 3 of Whalley et al. (2018)[42]
XYP (ppb)	0.05	Max noontime concentration during easterly flow in Table 3 of Whalley et al. (2018)[42]
ISO (ppb)	0.48	Max noontime concentration during easterly flow in Table 3 of Whalley et al. (2018)[42]
MACR (ppb)	0.12	Max noontime concentration during easterly flow in Table 3 of Whalley et al. (2018)[42]
MVK (ppb)	0.13	Max noontime concentration during easterly flow in Table 3

		of Whalley et al. (2018)[42]
HCHO (ppb)	29.9	Max noontime concentration during easterly flow in Table 3 of Whalley et al. (2018)[42]
ACD (ppb)	9.2	Max noontime concentration during easterly flow in Table 3 of Whalley et al. (2018)[42]
ALD (ppb)	0.16	Max noontime concentration during easterly flow in Table 3 of Whalley et al. (2018)[42]
ACT (ppb)	5.3	Max noontime concentration during easterly flow in Table 3 of Whalley et al. (2018)[42]
BALD (ppb)	0.06	Max noontime concentration during easterly flow in Table 3 of Whalley et al. (2018)[42]
KET (ppb)	0.84	Max noontime concentration during easterly flow in Table 3 of Whalley et al. (2018)[42]
EOH (ppb)	6.8	Max noontime concentration during easterly flow in Table 3 of Whalley et al. (2018)[42]
MOH (ppb)	8.9	Max noontime concentration during easterly flow in Table 3 of Whalley et al. (2018)[42]
ROH (ppb)	1.7	Max noontime concentration during easterly flow in Table 3 of Whalley et al. (2018)[42]
Solar zenith angle (°)	31.9	Calculated by TUV[a]
Photolysis frequencies (s^{-1})		
$O_3 \rightarrow O^1D$	1.53×10^{-5}	Max noontime value during 2012-07-24 to 2012-07-26 in Figure 1 of Whalley et al. (2018)[42]
$NO_2 \rightarrow NO + O$	8×10^{-3}	$j(NO_2)$, Max noontime value during 2012-07-24 to 2012-07-26 in Figure 8 of Malkin et al. (2016)[43]
$NO_2 \rightarrow NO + O$	8.5×10^{-3}	$j(NO_2)$_TUV, Calculated by TUV[a]
$HONO \rightarrow NO + OH$	1.48×10^{-3}	Calculated by TUV[a], scaled by $j(NO_2)/j(NO_2)$_TUV
$HCHO \rightarrow H_2 + CO$	2.54×10^{-5}	Calculated by TUV[a], scaled by $j(NO_2)/j(NO_2)$_TUV
$HCHO \rightarrow H + HCO$	3.95×10^{-5}	Calculated by TUV[a], scaled by $j(NO_2)/j(NO_2)$_TUV
Temperature (K)	300.6	Max noontime value during 2012-07-24 to 2012-07-26 in Figure 1 of Whalley et al. (2018)[42]
Pressure (hPa)	968.71	Minimum pressure in Table 1 of Valach et al. (2015)[44]
RH (%)	40	http://weather.uwyo.edu/, max noontime value during 2012-07-24 to 2012-07-26, London City Airport Station
OH (10^6 cm^{-3})	7.7	Max noontime value during 2012-07-24 to 2012-07-26 in Figure 2 of Whalley et al. (2018)[42]
k_{OH} (s^{-1})	21	Noontime value during 2012-07-24 to 2012-07-26 in Figure 1 of Whalley et al. (2016)[45]

Note that:

[a] Max noontime value during easterly flow (2012-07-24 to 2012-07-26), the latitude is 51.3126 °N and the longitude is 0.1248 °W[42].

Supplementary Table 8. The activation enthalpies for the H-migration of $R(CO)O_2$ radicals derived from acetaldehyde ($CH_3C(O)O_2$) under different methods.

Methods	Activation enthalpies for transition state (kcal mol^{-1})
W3X-L//CCSD(T)-F12a/cc-pVDZ-F12	29.30
W2X//CCSD(T)-F12a/cc-pVDZ-F12	29.59
WMS//M06-2X/MG3S	29.39
CCSD(T)-F12a/jun-cc-pVTZ//M06-2X/MG3S	29.48
CCSD(T)-F12a/cc-pVDZ-F12//M06-2X/MG3S	29.53

.**Supplementary Table 9.** The relative energies (kcal/mol) of distinguish conformers for reactant and transition states.

$R(C_6H_{11}O_3)$		TS5		TS4		TS2	
structures	Relative energies	structures	Relative energies	structures	Relative energies	structures	Relative energies
1	0.092025	1	0.000182	**1**	**0.000000**	**1**	**0.000000**
2	0.536567	2	0.000029	2	0.000735	2	0.001698
3	0.507323	**3**	**0.000000**	3	0.001527	3	0.001296
4	0.555868	4	0.001566	4	0.000760		
5	2.294800	5	0.000528	5	0.002054		
**6**	**0.00000**	6	0.000935	6	0.001400		
7	0.49538	7	0.002271	7	0.003152		
8	0.459814	8	0.001429	8	0.002268		
9	0.608492	9	0.001644				
10	0.607869	10	0.001391				
11	2.12493	11	0.001330				
12	1.27288	12	0.002840				
13	0.931321	13	0.001930				
14	1.39412	14	0.003220				
15	1.38061	15	0.004947				
16	1.30647	16	0.001521				
17	0.808449	17	0.001516				
18	2.5835	18	0.001459				
19	1.35445	19	0.002200				
20	3.2044	20	0.003185				
21	1.29774	21	0.002741				
22	0.855578						
23	1.26603						
24	1.3727						

25	0.644786						
26	0.744275						
27	3.02219						
28	1.84633						
29	1.73491						
30	1.29639						
31	1.8373						
32	1.77948						
33	0.949366						
34	0.732864						
35	1.17509						
36	1.14600						
37	1.19479						
38	2.93461						
39	0.530398						
40	1.02000						
41	0.98994						
42	1.07411						
43	1.06325						
44	2.68009						
45	1.32161						
46	1.52261						
47	1.97909						
48	1.97021						
49	1.90905						
50	1.78167						
51	3.56164						
52	1.93452						
53	3.77096						
54	1.88394						
55	1.22678						
56	1.6589						
57	1.73196						
58	1.10393						
59	1.17089						
60	3.46248						
61	1.91493						
62	1.96923						
63	1.14423						

Note: This table marked in bold font is the lowest energy structure, which is set to the energy zero, and the energy of other independent structures is obtained relative to the lowest energy structure. All conformers of the reactant (R) were optimized using M06-2X/MG3S method. Due to the higher barrier of

TS2, TS4, and TS5, they hardly effect the kinetics of the reaction. Thus, all conformers of transition states (TS2, TS4 and TS5) were optimized using the more economical B3LYP/6-31G (d, p) method. Then, all lowest energy conformers of reactant and transition states were reoptimized using M06-2X/MG3S method to obtain the zero-point vibrational energy correction.

Supplementary Table 10. Vibrational frequency (cm^{-1}) for all structures under different theoretical methods.

(a) calculated by CCSD(T)-F12a/cc-pVDZ-F12 method

Species	Vibrational frequency (cm^{-1})						
$CH_3C(O)OO$	118.43	152.05	336.89	504.53	534.45	563.06	761.40
	1002.58	1050.59	1136.02	1194.22	1409.21	1468.32	1475.77
	1881.09	3074.95	3151.92	3186.53			
TS	**1815.76i**	145.88	270.38	490.90	532.81	613.42	667.88
	840.93	904.68	1041.46	1067.34	1141.98	1173.91	1426.03
	1742.26	1879.24	3126.03	3214.51			
P	204.35	322.43	327.38	434.75	489.17	615.33	679.71
	746.44	872.48	999.09	1027.95	1289.60	1459.82	1510.84
	1731.97	3185.12	3314.08	3490.86			

(b) calculated by M06-2X/MG3S method

Note: Bold fonts with suffix i are the imaginary frequency of the transition state.

Species	Vibrational frequency (cm^{-1})						
$CH_3C(O)O_2$	140.21	200.14	348.10	516.75	551.54	584.76	
	784.68	1008.00	1055.79	1182.72	1281.22	1405.86	
	1467.57	1473.45	1950.29	3098.32	3166.94	3208.06	
TS	**1827.93i**	138.71	281.18	502.63	544.81	624.08	
	698.30	867.13	1042.44	1043.23	1060.65	1130.32	
	1193.27	1419.29	1767.45	1948.21	3144.17	3232.14	
P	199.67	330.02	331.51	414.80	439.24	620.52	
	697.34	758.01	909.27	1042.39	1102.86	1316.64	
	1461.73	1525.59	1780.85	3204.69	3328.28	3615.92	
$C_6H_{11}O_3$	56.44	62.63	86.76	132.06	140.26	152.35	222.45
	247.88	252.34	399.22	436.61	530.49	577.04	611.47
	730.21	762.48	835.10	853.63	920.91	974.65	
	1037.46	1074.74	1077.80	1096.90	1133.64	1148.63	1227.01
	1268.16	1282.33	1291.01	1332.56	1335.97	1344.89	1399.56
	1415.75	1421.11	1451.70	1489.69	1494.49	1505.97	1507.16
	1516.44	1942.61	3032.71	3051.37	3060.51	3061.42	3067.36
	3080.17	3086.73	3101.49	3119.89	3126.68	3137.27	
TS1	**1751.91i**	58.92	123.64	149.83	182.07	217.26	
	261.71	300.23	357.13	395.30	497.83	508.28	
	553.08	590.62	738.07	768.76	808.85	834.20	861.25
	924.68	955.85	1008.09	1072.00	1084.54	1112.80	1119.05

	1141.02 1170.18 1185.40 1239.76 1249.78 1299.94 1320.80<br>1362.18 1384.16 1407.63 1411.47 1454.38 1478.98 1490.45<br>1492.84 1495.81 1503.97 1896.80 3023.20 3040.81 3057.05<br>3076.46 3090.33 3111.29 3118.37 3126.96 3148.60 3182.49
TS2	**1731.10i** 40.67 91.92 141.85 170.46 241.67 262.97<br>295.04 388.48 404.00 473.48 512.30 582.08 621.67<br>731.84 764.84 780.31 864.05 895.37 927.01 955.89<br>1011.01 1060.14 1090.78 1097.83 1118.70 1133.09<br>1167.65 1186.04 1236.85 1275.19 1293.97 1313.13 1336.26<br>1378.19 1402.46 1419.56 1474.86 1484.15 1494.50 1499.27<br>1507.02 1511.33 1897.87 3016.19 3057.38 3064.43 3074.91<br>3109.67 3116.15 3128.28 3133.38 3147.59 3175.59
TS3	**1822.09i** 67.16 119.09 140.33 173.96 204.58 319.59<br>375.53 383.09 442.26 515.42 533.00 586.19 645.54<br>707.40 741.87 782.59 838.22 846.22 917.91 981.50<br>1002.42 1038.81 1076.67 1109.07 1116.87 1131.03 1147.40<br>1211.94 1229.75 1254.08 1278.62 1308.85 1355.50 1375.50<br>1388.57 1398.54 1437.15 1459.86 1475.03 1489.83 1500.46<br>1504.54 1891.31 3021.07 3050.72 3068.54 3071.75 3098.25<br>3106.79 3110.42 3118.14 3180.85 3185.76
TS4	**1734.13i** 56.83 71.49 87.02 149.33 167.23 245.49<br>247.86 308.15 361.25 461.60 503.80 567.08 605.93<br>696.90 740.19 805.42 830.18 867.12 915.84 975.01<br>1030.56 1077.71 1083.43 1095.74 1136.15 1139.36 1203.41<br>1241.78 1252.07 1260.93 1283.15 1319.09 1332.70 1380.95<br>1413.56 1419.78 1455.51 1472.84 1497.44 1507.16 1513.08<br>1585.65 1902.68 3006.77 3053.89 3060.01 3063.29 3074.06<br>3091.27 3105.86 3129.22 3133.15 3141.63
TS5	**1668.78i** 37.85 48.74 95.69 107.07 133.72 186.70<br>213.98 248.95 330.04 418.25 522.25 594.02 682.56<br>692.89 726.24 757.93 850.18 881.78 931.55 967.18<br>1034.26 1042.80 1072.92 1087.05 1135.45 1151.04 1178.49<br>1195.11 1241.24 1251.91 1308.61 1326.20 1336.16 1386.15<br>1411.05 1419.35 1471.91 1491.72 1501.00 1506.89 1515.01<br>1772.06 1941.05 3028.76 3040.58 3053.39 3062.20 3067.69<br>3082.48 3097.47 3128.59 3133.83 3139.71
P1	17.20 48.02 51.69 105.92 140.67 166.36 213.61<br>258.13 299.90 353.41 414.91 449.55 458.34 486.58<br>617.23 755.47 771.94 850.42 889.55 973.48 982.62<br>999.31 1038.12 1056.30 1090.74 1129.79 1152.60 1194.08<br>1225.46 1242.89 1304.40 1334.92 1356.11 1408.09 1416.33<br>1429.95 1467.68 1476.23 1481.93 1491.60 1502.94 1517.25<br>1844.89 2983.65 2997.80 3040.04 3064.70 3073.03 3080.04<br>3107.88 3126.94 3134.20 3172.29 3631.35

P2	13.03 35.73 54.43 77.85 109.52 201.37 211.00<br>240.53 284.60 370.10 384.41 405.72 428.48 502.19<br>606.00 741.94 756.24 784.61 920.80 980.08 998.39<br>1027.72 1053.17 1069.52 1086.19 1139.78 1155.44 1182.88<br>1220.05 1272.14 1279.13 1306.77 1352.78 1409.97 1415.82<br>1443.36 1466.38 1474.47 1484.27 1504.09 1509.70 1519.45<br>1847.05 2980.16 2994.29 3039.77 3063.60 3065.00 3075.72<br>3116.46 3131.40 3142.13 3166.80 3635.02
P3	13.17 59.38 90.11 99.06 134.27 160.90 217.02<br>264.31 293.57 366.06 413.80 419.26 476.09 511.16<br>615.93 737.62 753.30 828.90 849.70 905.39 977.74<br>989.46 1039.00 1061.37 1088.34 1111.97 1134.90 1197.12<br>1209.40 1236.03 1298.76 1316.44 1330.72 1371.84 1391.59<br>1420.38 1467.44 1468.87 1478.18 1493.42 1508.53 1517.09<br>1847.99 2991.77 3041.18 3061.13 3068.45 3073.78 3082.31<br>3105.44 3123.66 3166.04 3271.54 3631.98
P4	21.25 40.90 63.65 84.79 134.81 194.17 228.97<br>242.02 283.03 357.20 374.99 407.58 429.43 495.59<br>611.69 733.50 752.35 855.78 912.20 917.91 980.89<br>1027.75 1056.93 1079.41 1095.36 1139.84 1153.47 1189.14<br>1224.83 1251.92 1279.07 1322.89 1350.22 1403.79 1417.40<br>1444.67 1450.37 1473.86 1497.84 1505.82 1512.82 1518.38<br>1851.93 2964.11 2979.27 3020.87 3056.60 3060.99 3073.15<br>3091.80 3126.39 3137.58 3210.91 3635.51
P5	34.32 59.24 73.57 120.55 166.65 219.33 223.00<br>251.66 301.50 400.64 402.63 434.39 516.55 593.79<br>699.46 739.98 758.15 810.49 910.66 925.48 988.95<br>1002.55 1035.84 1075.28 1112.12 1122.85 1163.09 1221.79<br>1255.81 1274.08 1318.96 1334.19 1343.03 1391.93 1416.98<br>1459.65 1487.37 1492.07 1502.03 1505.90 1514.65 1523.73<br>1770.53 3046.13 3054.39 3061.78 3068.18 3077.95 3097.73<br>3120.98 3129.63 3137.31 3228.30 3612.25

Supplementary Table 11. Absolute energies in hartrees (1 hartree ≡ 1 a.u.)

Species	Methods	Total energies (a.u.)
$CH_3C(O)O_2$	W3X-L//CCSD(T)-F12a/cc-pVDZ-F12	-303.7759844
	W2X//CCSD(T)-F12a/cc-pVDZ-F12	-303.7734294
	WMS//M06-2X/MG3S	-303.7112443
	CCSD(T)-F12a/jun-cc-pVTZ//M06-2X/MG3S	-303.2472305
	CCSD(T)-F12a/cc-pVDZ-F12//M06-2X/MG3S	-303.2258527
	M06-2X/MG3S	-303.543425
TS	W3X-L//CCSD(T)-F12a/cc-pVDZ-F12	-303.6878984
	W2X//CCSD(T)-F12a/cc-pVDZ-F12	-303.6843439
	WMS//M06-2X/MG3S	-303.6582499
	CCSD(T)-F12a/jun-cc-pVTZ//M06-2X/MG3S	-303.1940885
	CCSD(T)-F12a/cc-pVDZ-F12//M06-2X/MG3S	-303.1726418
	M06-2X/MG3S	-303.486362
P	WMS//M06-2X/MG3S	-303.7064016
	M06-2X/MG3S	-303.537552
$C_6H_{11}O_3$	WMS//M06-2X/MG3S	-461.0068356
	M06-2X/MG3S	-460.76699
TS1	WMS//M06-2X/MG3S	-460.9740889
	M06-2X/MG3S	-460.735242
TS2	WMS//M06-2X/MG3S	-460.9711682
	M06-2X/MG3S	-460.731836
TS3	WMS//M06-2X/MG3S	-460.9666958
	M06-2X/MG3S	-460.728411
TS4	WMS//M06-2X/MG3S	-460.9675676
	M06-2X/MG3S	-460.726409
TS5	M06-2X/MG3S	-460.715719
P1	M06-2X/MG3S	-460.761787
P2	M06-2X/MG3S	-460.760542
P3	M06-2X/MG3S	-460.756236
P4	M06-2X/MG3S	-460.75968
P5	M06-2X/MG3S	-460.770459

Supplementary Table 12. Cartesian coordinates (Å) for all reactants and transition states are calculated.

(a) by CCSD(T)-F12a/cc-pVDZ-F12 method

Species	Coordinates (Å)			
$CH_3C(O)O_2$	C	-0.5472837715	0.1343811967	-0.0000018576
	O	-1.4797667421	0.8715784358	-0.0000060715
	C	-0.5321813765	-1.3609565398	-0.0000018102
	H	-1.5643378934	-1.6970387414	-0.0000042745

	H	-0.0050105421	-1.7311039463	-0.8772616845
	H	-0.0050144348	-1.7311042073	0.8772602506
	O	0.7367463172	0.7769653233	0.0000039579
	O	1.7467394433	-0.0807555210	0.0000064899
TS	C	0.1074891169	1.3458731048	-0.0187056285
	H	-1.1163153873	0.9806530913	-0.0403509646
	H	0.3482079524	1.8932090218	-0.9242817433
	H	0.3039176970	1.8938713082	0.8973074132
	C	0.5843008678	-0.0793880365	-0.0043770128
	O	-0.4849500030	-0.9363390581	-0.0023173767
	O	1.7087998615	-0.4755471434	0.0047358132
	O	-1.6850024654	-0.1449671081	-0.0253358105
P	C	0.4685429295	0.1289102180	-0.0000006248
	O	0.2183432333	1.3201666146	0.0000090735
	C	1.7754899264	-0.4841992210	-0.0000017314
	H	2.6369716088	0.1633739607	0.0000058395
	H	1.8752184156	-1.5581719324	-0.0000100672
	H	-1.5226455329	0.7684951619	0.0000063108
	O	-0.5126021178	-0.8114127035	-0.0000118428
	O	-1.7983924629	-0.1747030981	-0.0000049579

(b) by M06-2X/MG3S method

Species	Coordinates (Å)			
$CH_3C(O)O_2$	C	-0.54398000	0.13244000	0.00000000
	O	-1.46787700	0.86678400	0.00000400
	C	-0.53391600	-1.35931300	-0.00000200
	H	-1.56504000	-1.69407600	-0.00001200
	H	-0.00640500	-1.73085600	-0.87593500
	H	-0.00642400	-1.73085800	0.87594200
	O	0.73579700	0.76333200	-0.00000800
	O	1.73773600	-0.06548700	0.00000600
TS	C	-0.58264300	-0.07558000	0.00000000
	O	-1.69930000	-0.46687100	0.00000000
	C	-0.10058300	1.34727000	0.00000000
	H	-0.31006800	1.89748200	0.91049200
	H	1.13074300	0.96474600	0.00000000
	H	-0.31006800	1.89748200	-0.91049200
	O	0.48524300	-0.92129300	0.00000000
	O	1.66265100	-0.16056700	0.00000000
P	C	0.47521700	0.13536800	0.00000100
	O	0.23883500	1.32166800	-0.00000200
	C	1.77382700	-0.48517700	-0.00000200
	H	2.63859100	0.15600400	0.00002200
	H	1.86847900	-1.55826600	-0.00003000

	H	-1.56002400	0.76216100	0.00000000
	O	-0.51215000	-0.78938900	0.00000600
	O	-1.78184900	-0.18991000	-0.00000300
$C_6H_{11}O_3$	C	1.93363100	0.50842200	0.00000000
	O	2.00225900	1.68737600	0.00000000
	C	0.71317200	-0.35664200	0.00000000
	H	0.76473700	-1.01783300	-0.86756900
	H	0.76473700	-1.01783300	0.86756900
	O	3.19051100	-0.17040200	0.00000000
	O	3.09777900	-1.46729200	0.00000000
	C	-0.55795400	0.47962900	0.00000000
	H	-0.55920800	1.13584000	-0.87246400
	H	-0.55920800	1.13583900	0.87246400
	C	-1.80898200	-0.38994200	0.00000000
	H	-1.79837000	-1.04683700	0.87547000
	H	-1.79837000	-1.04683600	-0.87547100
	C	-3.09551400	0.42760400	0.00000000
	H	-3.10324600	1.08285900	-0.87457700
	H	-3.10324600	1.08285900	0.87457600
	C	-4.34260300	-0.44800400	0.00000000
	H	-4.36525300	-1.09141700	0.88052500
	H	-5.25221400	0.15092200	0.00000000
	H	-4.36525300	-1.09141700	-0.88052500
TS1	C	-1.61282600	-0.28607900	-0.13668400
	O	-2.48669500	-0.18715200	-0.93921100
	C	-1.23405300	0.76189200	0.87448900
	H	-2.15160500	1.27548200	1.14949000
	H	-0.79915200	0.31077300	1.76029200
	O	-0.81145100	-1.40535700	-0.23296900
	O	0.06310600	-1.57796300	0.81965000
	C	-0.25919300	1.76393800	0.21996400
	H	0.12389000	2.42307900	1.00048900
	H	-0.82183800	2.38427200	-0.47756200
	C	0.90602500	1.12016500	-0.53381800
	H	1.54495900	1.91525600	-0.93604300
	H	0.53211200	0.57736200	-1.40823200
	C	1.76355300	0.19405400	0.28977100
	H	0.96866100	-0.77142900	0.61017700
	H	2.01619900	0.59043400	1.27517800
	C	2.91016600	-0.46473500	-0.42884400
	H	3.64150400	0.28707900	-0.74136500
	H	3.42317400	-1.18648600	0.20370900
	H	2.56038300	-0.97745400	-1.32515100

TS2	C	1.53088100	0.39740800	-0.27284700
	O	2.10944000	0.74922400	-1.25218900
	C	1.54102600	-0.98697300	0.31672600
	H	2.38237200	-1.50664700	-0.13236700
	H	1.68339400	-0.92484200	1.39292000
	O	0.70895100	1.32193300	0.33349200
	O	0.13682600	0.87355000	1.50807900
	C	0.22748200	-1.74481300	0.00414500
	H	0.26261200	-2.69311500	0.53774100
	H	0.21548700	-1.97312800	-1.06473400
	C	-1.03709300	-0.97633700	0.35153100
	H	-0.62194700	-0.02582700	1.11099300
	H	-1.72757200	-1.50024800	1.01182800
	C	-1.71582000	-0.25009300	-0.78257300
	H	-2.14456900	-1.00651600	-1.45168800
	H	-0.96879400	0.28369500	-1.37617100
	C	-2.79807900	0.71312400	-0.31255900
	H	-2.36289800	1.49836000	0.30563900
	H	-3.29890100	1.18341200	-1.15662400
	H	-3.55131300	0.19330800	0.28087800
TS3	C	1.49427700	-0.02954900	-0.07346900
	O	2.28638000	-0.53935500	-0.80274500
	C	0.74108800	-0.75676500	1.01021000
	H	1.49253000	-1.26856900	1.60997200
	H	0.19571300	-0.07455800	1.65241800
	O	1.22074300	1.29956200	-0.30829500
	O	0.36215200	1.88422700	0.59847500
	C	-0.18609700	-1.79665700	0.36557000
	C	-1.21529700	-1.21309600	-0.60337100
	H	-1.78463700	-2.04446900	-1.01866900
	H	-0.69800200	-0.75235500	-1.45035400
	C	-2.18250200	-0.19415000	0.02571100
	H	-2.22631000	-0.33118600	1.10954600
	H	-3.19885600	-0.39661600	-0.33131900
	C	-1.89337500	1.24574100	-0.30271800
	H	-1.72651200	1.45416900	-1.35800600
	H	-2.54703700	1.97569700	0.16904500
	H	-0.73655800	1.60441800	0.18790200
	H	0.42918800	-2.52274300	-0.16599900
	H	-0.70228900	-2.33240600	1.16438600
TS4	C	2.25773800	-0.29072000	0.00511700
	O	3.30714100	-0.80640100	-0.20395100
	C	0.95820900	-0.99371300	0.33856500
	H	1.09977200	-2.03919900	0.06720900

	H	0.81548700	-0.93486700	1.42001900
	O	2.16072800	1.07140000	-0.13582900
	O	0.98629000	1.60887900	0.36664500
	C	-0.21545000	-0.35060100	-0.35959700
	H	-0.17877700	-0.42315900	-1.44731700
	H	0.12475200	0.86992400	-0.14348000
	C	-1.58000200	-0.55200200	0.23795000
	H	-1.55248400	-0.28863700	1.29968000
	H	-1.82347700	-1.62213700	0.19941500
	C	-2.67452700	0.24137800	-0.46813300
	H	-2.68959600	-0.03241100	-1.52568000
	H	-2.42632900	1.30449300	-0.42694700
	C	-4.04717400	0.00422200	0.14841200
	H	-4.05625400	0.29505600	1.19944000
	H	-4.81727000	0.57839200	-0.36400700
	H	-4.32187000	-1.04986700	0.09286600
TS5	C	-2.12419500	0.50831200	0.08024500
	O	-2.52000700	1.62237600	-0.00618300
	C	-0.79122300	0.03994900	0.60400600
	H	-0.69984900	0.15036500	1.68336600
	H	-1.09006800	-1.17323400	0.38489600
	O	-2.86355200	-0.55716400	-0.33003900
	O	-2.12681500	-1.73117400	-0.11715000
	C	0.41410100	0.47416200	-0.18540300
	H	0.46944200	1.56764900	-0.13058700
	H	0.27020300	0.22670900	-1.23989000
	C	1.71199800	-0.13751700	0.33109800
	H	1.64235900	-1.22811100	0.28691800
	H	1.84009200	0.12297100	1.38591400
	C	2.93071200	0.32535500	-0.45860100
	H	2.99374200	1.41545600	-0.41413900
	H	2.79465600	0.06709800	-1.51162100
	C	4.22470400	-0.28916700	0.06054800
	H	4.18945900	-1.37751800	-0.00049100
	H	5.08558000	0.05041100	-0.51326700
	H	4.39078500	-0.02066300	1.10452600
P1	C	1.59693500	0.08777500	-0.08261700
	O	2.00059900	1.05823800	-0.66590100
	C	0.17261800	-0.23311500	0.25935100
	H	-0.19318100	-0.91745900	-0.51139500
	H	0.15008700	-0.78649600	1.19884800
	O	2.42714800	-0.88374000	0.33574800
	O	3.76526300	-0.62701200	-0.02225500
	C	-0.69820900	1.01627500	0.31701400

	H	-0.61379200	1.55340500	-0.62872500
	H	-0.31908500	1.68608600	1.08967800
	C	-2.16116500	0.67028300	0.59054000
	H	-2.71737600	1.60596500	0.74403900
	H	-2.24425200	0.12502000	1.53727600
	C	-2.79253200	-0.12495200	-0.49920200
	H	-2.50481400	0.10221000	-1.51912400
	C	-4.05200000	-0.87741900	-0.26065300
	H	-4.28576600	-1.55105100	-1.08334300
	H	-3.99170900	-1.46505000	0.65831100
	H	-4.91018700	-0.20275700	-0.14107700
	H	3.69211100	0.23715700	-0.47183000
P2	C	-1.76929000	0.08321700	-0.02945800
	O	-1.97636700	1.26620800	-0.06730700
	C	-0.43435200	-0.60298300	-0.02377100
	H	-0.43568100	-1.34760800	0.77384600
	H	-0.34488000	-1.15772700	-0.96041300
	O	-2.77079700	-0.81280800	0.00450100
	O	-4.03658000	-0.19489100	-0.02647600
	C	0.71574100	0.38245600	0.13461400
	H	0.56929700	0.95174500	1.06297500
	H	0.66732300	1.12983400	-0.66226200
	C	2.04208700	-0.29046800	0.13029900
	H	-3.79969600	0.75221000	-0.05448100
	H	2.10573200	-1.32318400	0.45491500
	C	3.29570000	0.50495700	0.04158400
	H	3.20554200	1.23254100	-0.77201400
	H	3.41739300	1.10984700	0.95189100
	C	4.53815900	-0.35641600	-0.16091400
	H	4.46053600	-0.93279200	-1.08265700
	H	5.43920300	0.25231400	-0.21541000
	H	4.65691400	-1.05983100	0.66374200
P3	C	-1.61494200	0.05114900	-0.12615600
	O	-1.71162400	1.13513000	-0.63521200
	C	-0.38033300	-0.79768000	-0.03155300
	H	-0.39182700	-1.32530300	0.92284400
	H	-0.46537300	-1.56340600	-0.80786300
	O	-2.66987700	-0.56683600	0.43352400
	O	-3.85235700	0.18937300	0.31227800
	C	0.89267100	0.01669800	-0.21511100
	H	0.95266700	0.78052300	0.56290000
	H	0.84262600	0.55204000	-1.16485500
	C	2.13777000	-0.86163200	-0.17808300
	H	2.07321300	-1.61784200	-0.96430700

	H	2.17843800	-1.40218400	0.77258400
	C	3.42793400	-0.05545400	-0.34967500
	H	3.39223800	0.49375900	-1.29382800
	C	3.67950100	0.88225800	0.77641500
	H	4.20979100	1.81007900	0.62957100
	H	3.49086400	0.56552700	1.79291400
	H	4.26615600	-0.75875700	-0.44593000
	H	-3.53354100	0.99218400	-0.14377600
P4	C	1.78403200	0.09109200	0.11359900
	O	1.95207200	1.27428100	0.22773900
	C	0.50148500	-0.66753700	0.31110500
	H	0.49714900	-1.53628900	-0.35243100
	H	0.55233100	-1.08398200	1.32907100
	O	2.78880400	-0.74490400	-0.19715300
	O	4.01810400	-0.06990100	-0.32773600
	C	-0.70327300	0.18308900	0.12970000
	H	-0.58563500	1.25569500	0.19690800
	H	3.75770600	0.85891700	-0.17452800
	C	-2.05607900	-0.42821700	0.18064500
	H	-2.26809300	-0.79226700	1.19826800
	H	-2.08255400	-1.32504200	-0.45075300
	C	-3.16882000	0.52726000	-0.24190300
	H	-3.13803100	1.41141000	0.39891000
	H	-2.97060600	0.87449900	-1.25800600
	C	-4.54653800	-0.11813900	-0.16982900
	H	-4.76870500	-0.44866400	0.84574800
	H	-5.32867600	0.57574600	-0.47427700
	H	-4.60156100	-0.99112500	-0.82160900
P5	C	-1.58791900	-0.10151300	0.12894200
	O	-1.44122600	0.84168400	0.87699700
	C	-0.62419500	-1.13425800	-0.15009700
	H	-0.89027600	-1.89421400	-0.86999500
	H	-3.18866900	1.27960300	0.35662700
	O	-2.73976300	-0.28900600	-0.55871100
	O	-3.67563000	0.72218200	-0.28212300
	C	0.71694700	-1.06918400	0.47210800
	H	1.15506700	-2.06671700	0.52087800
	H	0.62387200	-0.68440700	1.48968200
	C	1.66133400	-0.13634900	-0.31175500
	H	1.21325100	0.85906600	-0.35281300
	H	1.74915800	-0.49000200	-1.34237500
	C	3.04359900	-0.05545700	0.32480400
	H	2.94169500	0.29386700	1.35499300
	H	3.47537300	-1.05809900	0.37831100

	C	3.97820400	0.86964100	-0.44524300
	H	3.57384000	1.88153000	-0.48671800
	H	4.96147400	0.92041600	0.02039400
	H	4.11034700	0.52279800	-1.47083900

Supplementary References

1. Hofzumahaus A, *et al.* Amplified Trace Gas Removal in the Troposphere. *Science* **324**, 1702-1704 (2009).
2. Lu KD, *et al.* Missing OH source in a suburban environment near Beijing: observed and modelled OH and HO2 concentrations in summer 2006. *Atmospheric Chemistry and Physics* **13**, 1057-1080 (2013).
3. Lu KD, *et al.* Observation and modelling of OH and HO2 concentrations in the Pearl River Delta 2006: a missing OH source in a VOC rich atmosphere. *Atmospheric Chemistry and Physics* **12**, 1541-1569 (2012).
4. Ma XF, *et al.* OH and HO2 radical chemistry at a suburban site during the EXPLORE-YRD campaign in 2018. *Atmospheric Chemistry and Physics* **22**, 7005-7028 (2022).
5. Tan Z, *et al.* Radical chemistry at a rural site (Wangdu) in the North China Plain: observation and model calculations of OH, HO2 and RO2 radicals. *Atmospheric Chemistry and Physics* **17**, 663-690 (2017).
6. Tan Z, *et al.* Experimental budgets of OH, HO2, and RO2 radicals and implications for ozone formation in the Pearl River Delta in China 2014. *Atmospheric Chemistry and Physics* **19**, 7129-7150 (2019).
7. Yang X, *et al.* Radical chemistry in the Pearl River Delta: observations and modeling of OH and HO2 radicals in Shenzhen in 2018. *Atmospheric Chemistry and Physics* **22**, 12525-12542 (2022).
8. Yang XP, *et al.* Observations and modeling of OH and HO2 radicals in Chengdu, China in summer 2019. *Science of the Total Environment* **772**, (2021).
9. Fuchs H, *et al.* OH reactivity at a rural site (Wangdu) in the North China Plain: contributions from OH reactants and experimental OH budget. *Atmospheric Chemistry and Physics* **17**, 645-661 (2017).
10. Lou S, *et al.* Atmospheric OH reactivities in the Pearl River Delta - China in summer 2006: measurement and model results. *Atmospheric Chemistry and Physics* **10**, 11243-11260 (2010).
11. Peeters J, Muller J-F, Stavrakou T, Vinh Son N. Hydroxyl Radical Recycling in Isoprene Oxidation Driven by Hydrogen Bonding and Hydrogen Tunneling: The Upgraded LIM1 Mechanism. *Journal of Physical Chemistry A* **118**, 8625-8643 (2014).
12. Peeters J, Nguyen TL, Vereecken L. HOx radical regeneration in the oxidation of isoprene. *Physical Chemistry Chemical Physics* **11**, 5935-5939 (2009).
13. Goliff WS, Stockwell WR, Lawson CV. The regional atmospheric chemistry mechanism, version 2. *Atmos Environ* **68**, 174-185 (2013).
14. Lu KD, *et al.* Exploring atmospheric free-radical chemistry in China: the self-cleansing capacity and the formation of secondary air pollution. *Natl Sci Rev* **6**, 579-594 (2019).
15. Yang X, *et al.* Observations and modeling of OH and HO2 radicals in Chengdu, China in summer 2019. *The Science of the total environment* **772**, 144829-144829 (2021).
16. Adler TB, Knizia G, Werner H-J. A simple and efficient CCSD(T)-F12 approximation. *Journal of Chemical Physics* **127**, (2007).
17. Chan B, Radom L. W2X and W3X-L: Cost-Effective Approximations to W2 and W4 with kJ mol(-1) Accuracy. *Journal of Chemical Theory and Computation* **11**, 2109-2119

(2015).

18. Zhao Y, Xia L, Liao X, He Q, Zhao MX, Truhlar DG. Extrapolation of high-order correlation energies: the WMS model. *Physical Chemistry Chemical Physics* **20**, 27375-27384 (2018).
19. Zhao Y, Truhlar DG. The M06 suite of density functionals for main group thermochemistry, thermochemical kinetics, noncovalent interactions, excited states, and transition elements: two new functionals and systematic testing of four M06-class functionals and 12 other functionals. *Theoretical Chemistry Accounts* **120**, 215-241 (2008).
20. Lynch BJ, Zhao Y, Truhlar DG. Effectiveness of diffuse basis functions for calculating relative energies by density functional theory. *Journal of Physical Chemistry A* **107**, 1384-1388 (2003).
21. Bao JL, Truhlar DG. Variational transition state theory: theoretical framework and recent developments. *Chemical Society Reviews* **46**, 7548-7596 (2017).
22. Eckart C. The penetration of a potential barrier by electrons. *Physical Review* **35**, 1303-1309 (1930).
23. Eyring H. The activated complex in chemical reactions. *Journal of Chemical Physics* **3**, 107-115 (1935).
24. Zheng J, Truhlar DG. Quantum Thermochemistry: Multistructural Method with Torsional Anharmonicity Based on a Coupled Torsional Potential. *Journal of Chemical Theory and Computation* **9**, 1356-1367 (2013).
25. Viegas LP, Jensen F. A computer-based solution to the oxidation kinetics of fluorinated and oxygenated volatile organic compounds. *Environmental Science-Atmospheres* **3**, 855-871 (2023).
26. Zheng J, Mielke SL, Clarkson KL, Truhlar DG. <i>MSTor</i>: A program for calculating partition functions, free energies, enthalpies, entropies, and heat capacities of complex molecules including torsional anharmonicity. *Computer Physics Communications* **183**, 1803-1812 (2012).
27. Zheng J, Mielke SL, Bao JL, Meana-Pafeda R, Clarkson KL, Truhlar DG. MSTor computer program, version 2017-B. *University of Minnesota, Minneapolis, MN*, (2017).
28. Stephens PJ, Devlin FJ, Chabalowski CF, Frisch MJ. Ab Initio Calculation of Vibrational Absorption and Circular Dichroism Spectra Using Density Functional Force Fields. *The Journal of Physical Chemistry* **98**, 11623-11627 (1994).
29. Gonzalez C, Schlegel HB. An improved algorithm for reaction path following. *The Journal of Chemical Physics* **90**, 2154-2161 (1989).
30. Fukui K. THE PATH OF CHEMICAL-REACTIONS - THE IRC APPROACH. *Accounts of Chemical Research* **14**, 363-368 (1981).
31. Gonzalez C, Schlegel HB. REACTION-PATH FOLLOWING IN MASS-WEIGHTED INTERNAL COORDINATES. *Journal of Physical Chemistry* **94**, 5523-5527 (1990).
32. Pan S, Wang L. Atmospheric Oxidation Mechanism of m-Xylene Initiated by OH Radical. *Journal of Physical Chemistry A* **118**, 10778-10787 (2014).
33. Pan S-S, Wang L-M. The Atmospheric Oxidation Mechanism of o-Xylene Initiated by Hydroxyl Radicals. *Acta Physico-Chimica Sinica* **31**, 2259-2268 (2015).

34. Wang S, Wu R, Berndt T, Ehn M, Wang L. Formation of Highly Oxidized Radicals and Multifunctional Products from the Atmospheric Oxidation of Alkylbenzenes. *Environmental Science & Technology* **51**, 8442-8449 (2017).
35. Wu R, Pan S, Li Y, Wang L. Atmospheric Oxidation Mechanism of Toluene. *Journal of Physical Chemistry A* **118**, 4533-4547 (2014).
36. Bianchi F*, et al.* Highly Oxygenated Organic Molecules (HOM) from Gas-Phase Autoxidation Involving Peroxy Radicals: A Key Contributor to Atmospheric Aerosol. *Chemical Reviews* **119**, 3472-3509 (2019).
37. Liu Z, Vinh Son N, Harvey J, Mueller J-F, Peeters J. The photolysis of alpha-hydroperoxycarbonyls. *Physical Chemistry Chemical Physics* **20**, 6970-6979 (2018).
38. Rohrer F*, et al.* Maximum efficiency in the hydroxyl-radical-based self-cleansing of the troposphere. *Nature Geoscience* **7**, 559-563 (2014).
39. Wang S-n, Wu R-r, Wang L-m. Role of Hydrogen Migrations in Carbonyl Peroxy Radicals in the Atmosphere. *Chinese Journal of Chemical Physics* **32**, 457-466 (2019).
40. Atkinson R. Atmospheric reactions of alkoxy and beta-hydroxyalkoxy radicals. *International Journal of Chemical Kinetics* **29**, 99-111 (1997).
41. Atkinson R. GAS-PHASE TROPOSPHERIC CHEMISTRY OF ORGANIC-COMPOUNDS. *Journal of Physical and Chemical Reference Data*, R1-& (1994).
42. Whalley LK*, et al.* Understanding in situ ozone production in the summertime through radical observations and modelling studies during the Clean air for London project (ClearfLo). *Atmospheric Chemistry and Physics* **18**, 2547-2571 (2018).
43. Malkin TL*, et al.* Assessing chemistry schemes and constraints in air quality models used to predict ozone in London against the detailed Master Chemical Mechanism. *Faraday Discussions* **189**, 589-616 (2016).
44. Valach AC, Langford B, Nemitz E, MacKenzie AR, Hewitt CN. Seasonal and diurnal trends in concentrations and fluxes of volatile organic compounds in central London. *Atmospheric Chemistry and Physics* **15**, 7777-7796 (2015).
45. Whalley LK*, et al.* Atmospheric OH reactivity in central London: observations, model predictions and estimates of in situ ozone production. *Atmospheric Chemistry and Physics* **16**, 2109-2122 (2016).
